# Novel Broad-Spectrum Antiviral Inhibitors Targeting Host Factors Essential for Replication of Pathogenic RNA Viruses

**DOI:** 10.3390/v12121423

**Published:** 2020-12-10

**Authors:** Marianna Tampere, Aleksandra Pettke, Cristiano Salata, Olov Wallner, Tobias Koolmeister, Armando Cazares-Körner, Torkild Visnes, Maria Carmen Hesselman, Elena Kunold, Elisee Wiita, Christina Kalderén, Molly Lightowler, Ann-Sofie Jemth, Janne Lehtiö, Åsa Rosenquist, Ulrika Warpman-Berglund, Thomas Helleday, Ali Mirazimi, Rozbeh Jafari, Marjo-Riitta Puumalainen

**Affiliations:** 1 Science for Life Laboratory, Department of Oncology-Pathology, Karolinska Institutet, 171 65 Stockholm, Sweden; marianna.tampere@scilifelab.se (M.T.); aleksandra.pettke@scilifelab.se (A.P.); olov.wallner@scilifelab.se (O.W.); tobias.koolmeister@scilifelab.se (T.K.); a.cazaresk@gmail.com (A.C.-K.); torkild.visnes@sintef.no (T.V.); hesselman.maria@virology.uzh.ch (M.C.H.); elena.kunold@googlemail.com (E.K.); elisee.wiita@scilifelab.se (E.W.); christina.kalderen@scilifelab.se (C.K.); molly.lightowler@mmk.su.se (M.L.); annsofie.jemth@scilifelab.se (A.-S.J.); janne.lehtio@ki.se (J.L.); asarosenquist1@gmail.com (Å.R.); ulrika.warpmanberglund@scilifelab.se (U.W.-B.); thomas.helleday@scilifelab.se (T.H.); rozbeh.jafari@scilifelab.se (R.J.); 2 National Veterinary Institute, SE-756 51 Uppsala, Sweden; Ali.Mirazimi@ki.se; 3 Department of Microbiology, Public Health Agency of Sweden, 171 65 Stockholm, Sweden; cristiano.salata@unipd.it; 4 Department of Molecular Medicine, University of Padova, 35121 Padova, Italy; 5 Unit of Clinical Microbiology, Department of Laboratory Medicine, Karolinska Institute and Karolinska University Hospital, 17177 Stockholm, Sweden

**Keywords:** antivirals, virus-host interactions, pathogenic RNA viruses, HSP70, target identification

## Abstract

Recent RNA virus outbreaks such as Severe acute respiratory syndrome coronavirus 2 (SARS-CoV-2) and Ebola virus (EBOV) have caused worldwide health emergencies highlighting the urgent need for new antiviral strategies. Targeting host cell pathways supporting viral replication is an attractive approach for development of antiviral compounds, especially with new, unexplored viruses where knowledge of virus biology is limited. Here, we present a strategy to identify host-targeted small molecule inhibitors using an image-based phenotypic antiviral screening assay followed by extensive target identification efforts revealing altered cellular pathways upon antiviral compound treatment. The newly discovered antiviral compounds showed broad-range antiviral activity against pathogenic RNA viruses such as SARS-CoV-2, EBOV and Crimean-Congo hemorrhagic fever virus (CCHFV). Target identification of the antiviral compounds by thermal protein profiling revealed major effects on proteostasis pathways and disturbance in interactions between cellular HSP70 complex and viral proteins, illustrating the supportive role of HSP70 on many RNA viruses across virus families. Collectively, this strategy identifies new small molecule inhibitors with broad antiviral activity against pathogenic RNA viruses, but also uncovers novel virus biology urgently needed for design of new antiviral therapies.

## 1. Introduction

In the last decade, we have seen outbreaks of several pathogenic RNA viruses, like Ebola virus (EBOV), Crimean-Congo hemorrhagic fever virus (CCHFV) and Severe acute respiratory syndrome coronavirus 2 (SARS-CoV-2). EBOV and CCHFV both cause viral hemorrhagic fever with the average case fatality rate around 50–70% [1,2,3] and 5–30% [4], respectively. SARS-CoV-2 infection is mild or asymptomatic in the majority of cases; however, up to 10% of cases lead to severe respiratory distress, sepsis and may be lethal. The ongoing Coronavirus disease 2019 (COVID-19) pandemic caused by SARS-CoV-2 has led to 1,200,000 deaths as of November 2020 and unseen social and economic disruption worldwide. Common human coronaviruses such as Coronavirus 229E (CoV 229E) cause mild upper respiratory tract infections and around 15–30% of common cold cases in adults [5]. While RNA viruses cause a variety of diseases, a common denominator is the lack of antivirals to treat them, highlighting the need for the development of antivirals to tackle these diseases.

Hallmarks of RNA viruses include a heavy reliance on the host cell for viral replication [6] and high mutation rates [7,8] resulting in fast resistance against antiviral drugs targeting virus proteins [9,10,11]. Of note, coronaviruses are an exception to the high mutation rates of RNA viruses, as they have a proofreading mechanism [12,13]. Given that several host processes such as translation [14,15] and protein folding [16] are commonly exploited by RNA viruses across virus families, targeting host factors required by the virus is an attractive strategy to battle emerging RNA viruses. Host cells experience higher genetic barriers to evade host-targeting antivirals and drug resistance is much less likely to occur [17]. However, it is challenging to find druggable host proteins as targets for antiviral therapies with limited understanding of host cell–virus interactions. Unlike target-based drug discovery, phenotypic assays enable discovery of new classes of compounds without prior knowledge on the target or its role in the virus life cycle. Combining phenotypic screenings with target identification methods can lead to a discovery of novel antiviral targets as well as host–virus interactions.

During recent years, advanced mass-spectrometry-based target identification method thermal proteome profiling (TPP) has been developed to monitor drug–target interactions in living cells on a proteome-wide scale [18]. TPP measures changes of protein thermal stability upon compound binding to cellular targets and can be used to detect drug targets and changes in cellular pathways [18].

One of the pathways activated by RNA viruses is the oxidative stress pathway in the host cell [19,20,21]. The Helleday Laboratory has previously developed small molecule inhibitors targeting oxidative stress and nucleotide metabolism pathways in the context of cancer and inflammation [22,23]. Based on this background, we hypothesized that disturbing oxidative balance of the host cell can influence viral replication.

Here, we established an image-based phenotypic high-throughput screening assay coupled with automated image analysis and tested a set of in-house inhibitors targeting oxidative stress and nucleotide metabolism pathways. We identified novel small molecule compounds with antiviral activity against several pathogenic RNA viruses such as SARS-CoV-2, EBOV and CCHFV, demonstrating broad-activity of the hit compound. Lastly, we studied the pharmacological activity of the new antiviral compound using TPP showing its multifaceted effects on cellular HSP70 pathways.

## 2. Materials and Methods

### 2.1. Biosafety

All procedures in presence of infectious viruses were carried out under respective biosafety laboratory conditions according to Swedish Work and Health Authorities. Experiments using Hazara virus (HAZV) and CoV 229E were performed under BSL2, SARS-CoV-2 under BSL3 at Karolinska Institutet. CCHFV and EBOV under BSL4 conditions at the Public Health Agency of Sweden.

### 2.2. Cells and Viruses

Vero, Vero E6 and U87 cells (from ATCC, Manassas VA, USA) were cultured in complete Dulbecco’s modified Eagle medium (DMEM, Gibco, USA), U-2 OS (from ATCC, Manassas, VA, USA) in DMEM GlutaMAX™ medium (Gibco, USA), SW-13 (ATCC, Manassas, VA, USA) in Leibovitz L-15 (Gibco, USA) medium, HEK293T cells (ATCC, Manassas, VA, USA) in DMEM GlutaMAX™ medium, Huh7 cells (from ATCC, Manassas, VA, USA) in DMEM (Gibco, USA). Culture medium of all cell lines were supplemented with 10% (*v*/*v*) FBS (Thermo Fisher Scientific, Waltham, MA, USA), 50 U/mL penicillin and 50 μg/mL streptomycin (PS, Thermo Fisher Scientific, Waltham, MA, USA). All cell lines were maintained at 37 °C under 5% CO_2_, except SW-13 cells which were maintained under atmospheric CO_2_. All cell lines were routinely tested for Mycoplasma using a luminescence-based MycoAlert kit (Lonza, Basel, Switzerland).

HAZV (isolate JC280; from European Virus Archive, Marseille, France) stocks were amplified in SW13 cells. Virus titers were determined in Vero cells by focus-forming unit (FFU) assay combined with high-throughput immunofluorescence imaging of viral protein staining. CCHFV (IbAr 10200) and EBOV (Sudan Ebolavirus) were both from the Public Health Agency of Sweden. Stocks were grown on Vero cells and titers were determined by FFU in Vero cells, as previously described in [24]. CoV 229E (VR-740; from ATCC, Manassas, VA, USA) stocks were amplified in Huh7 cells. SARS-CoV-2 (isolate MT093571.1; from the Public Health Agency of Sweden) [25] were amplified in Vero E6 cells.

### 2.3. Virus Infections and Compound Treatments

Cells were infected with a MOI of 0.1, 0.5, 1 or 10 in 50 μL corresponding cell culture medium for 1 h. At least three wells of cells were left uninfected as a mock control. Virus-containing medium was removed and replaced with 100 μL dimethyl sulfoxide (DMSO) (Sigma-Adrich, St. Louis, MO, USA, max. 0.2%) or compound-containing medium in at least technical duplicates. Number of infected cells, cytotoxicity and progeny virus yield in supernatants were determined after 24–48 h post infection (hpi) by immunofluorescence assay.

Infections with CCHFV and EBOV were performed in 24-well plates. Vero cells were seeded at the density of 1 × 10^5^ cells per well. After 24 h cells were infected with the appropriate MOI in 1 mL of plenty medium for 1 h. Then, virus-containing medium was removed and replaced with 1 mL DMSO (max. 0.5%) or compound-containing medium. Each condition was tested in duplicates. At 48 hpi supernatants were collected and viral particles quantified by end-point dilution assay using immunostaining and plaques were counted manually.

### 2.4. Synthesis of Compounds

Details of compound synthesis are found in the Supplementary Material document.

### 2.5. Phenotypic Antiviral Screening

Compounds were dispersed in 384-well plates at final concentration of 10 μM, (max DMSO 0.1% *v*/*v*) and stored at 4 °C. SW13 cells were inoculated with HAZV in L15 medium in presence of 10% FBS and 1% PS for 1 h. After inoculation, the virus-containing medium was removed and washed with PBS (Gibco, USA). Cells were trypsinized and 3000 cells were seeded on 384-well plates containing screening compounds. Cells were incubated for 24 h. Infected cells were fixed in 4% paraformaldehyde (Santa Cruz, Dallas, TX, USA) in PBS for 20 min and in 1:1 (*v*/*v*) methanol-acetone for 20 min in −20 °C. Number of infected cells were quantified by high-throughput microscopy. Data were normalized to infected DMSO-treated controls.

### 2.6. Determining Antiviral and Cytotoxicity EC_50_

Compounds were dispersed in deep-well 96-well plates (Biotix, San Diego, CA, USA #DP-1200-9CUS) using the D300e Digital Dispenser (Tecan, Männedorf, Switzerland) and stored at 4 °C. Compound 1:2 dilution steps between 50 μM and 1.526 μM (final concentration; DMSO 0.5% *v*/*v*) were chosen. Compounds were dissolved in culture media. 5000 SW13 cells were seeded into 96-well plates and allowed to attach for 24 h. Cells were inoculated with HAZV in L15 medium in presence of 10% FBS and 1% PS for 1 h. After inoculation, virus-containing medium was removed and replaced with medium containing DMSO or compounds and cells were incubated for 24 h. Cells were fixed in 4% paraformaldehyde in PBS for 20 min and in 1:1 (*v*/*v*) methanol-acetone for 20 min in −20 °C and processed for high-throughput microscopy.

### 2.7. Virus Infectivity Assay

Cells were seeded in 96-well plates (Black 96-well microplate, flat bottom, Falcon). At 24 h after seeding, cells were inoculated with virus in 50 μL corresponding cell culture medium for 1 h. At least three wells of cells were left uninfected as a mock control. After 1 h virus inoculation, the virus-containing medium was removed and replaced with 100 μL DMSO (max. 0.5% *v*/*v*) or compound-containing medium in at least technical duplicates. At 24 hpi, virus-containing supernatants were collected and used in end-point dilution assay. Cells were fixed in 4% paraformaldehyde in PBS for 20 min and in 1:1 (*v*/*v*) methanol-acetone for 20 min in −20 °C and processed for high-throughput microscopy.

### 2.8. End-Point Dilution Assay by Immunofluorescence

To quantify viral titer, cells were seeded in 96-well plates (Falcon® 96-well Black/Clear Flat Bottom TC-treated Imaging Microplate, Corning, Glendale, AZ, USA) at a density of 5 × 10^3^ cells per well. 24 h later virus-containing supernatant samples were serially diluted on cells by 10-fold and incubated for 24 h. Supernatants were discarded, cells were fixed in 4% paraformaldehyde in PBS for 20 min and in 1:1 (*v*/*v*) methanol-acetone for 20 min in −20 °C and processed for high-throughput microscopy.

### 2.9. High-Throughput Microscopy

Immunofluorescence staining. Methanol-acetone was removed, plates were washed twice with PBS and incubated with virus-specific primary antibody (see antibody details in Table S2) in 0.2% BSA 0.1% Triton-X in PBS overnight at 4 °C. Primary antibody was removed and cells were washed 3 × 10 min with PBS and incubated with secondary antibody (donkey-anti-mouse Alexa-488; 1:800) and DAPI (1:1000, see details in Tables S2 and S3) in 0.2% BSA 0.1% Triton-X in PBS for 1 h. Cells were washed 3 × 10 min with PBS and retained in 100 μL PBS for image acquisition.

Automated image acquisition. Images were automatically captured with ImageXpress Micro XLS Widefield High-Content Analysis System (Molecular Devices, San Jose, CA, USA) typically using two fluorescent channels: DAPI (387/447) and FITC (472/520). Nine images were captured per each well using ×10 Plan Fluor 0.3 NA objective. Images were saved as 16-bit, gray-scale Tiff files, along with metadata in an Oracle database for further analysis.

Quantitative image analysis by CellProfiler. Images were loaded into open-source software CellProfiler (Broad Institute, Cambridge, MA, USA) and analysed by pipeline. Automated analysis had 4 main steps: Identifying primary objects, identifying secondary objects, measuring object intensity and classifying objects. Identifying primary objects were performed on DAPI stained images based on intensity threshold between the foreground and background and referred as “primary objects”. Secondary objects were identified based on the primary objects by expanding the area around each nucleus by 15 pixels and measuring intensity of this area from the FITC channel containing virus-specific staining. Classification based on nuclei expansion is possible given that virus replication is perinuclear. Upon identifying primary and secondary objects, object intensities were measured. Finally, based on secondary object mean intensity, cells were divided into two custom-defined bins: uninfected or infected. The threshold between two bins depends on staining quality, background and foreground intensity and is therefore defined at each CellProfiler analysis based on positive (infected cells) and negative (uninfected cells) controls. Data from object classification, shape, size and intensity measurements were exported to a spreadsheet.

### 2.10. Z-Factor Calculations

Z-factors were calculated and interpreted as described previously [26]. Briefly, the main signal of negative controls plus three times the standard deviation (SD) of those valuesx was set as a threshold for negative controls. Then, the mean signal of positive controls minus three times the SD of those values was set as the threshold for positive controls. The difference between the thresholds was calculated and was divided by the absolute value of the difference of thresholds. In infection assays uninfected DMSO-treated cells were used as negative control and infected DMSO-treated cells as positive control. In viability assays, media-only was used as negative control and DMSO treated as positive control.

### 2.11. Cell Viability Assays

Resazurin assay. Cells were seeded into 96-well plates and allowed to attach for 24 h. Respective media was exchanged for compound-containing with a stable DMSO concentration of maximally 0.5% throughout the plate and cells were incubated for 24, 48 or 72 h. After respective incubation times, 10 μg/mL Resazurin (Sigma-Aldrich, St. Louis, MO, USA, R7017, stock at 1 mg/mL in PBS) was added on top of cells and incubated between 1 to 4 h. Fluorescence was measured at 544/590 (excitation/emission) using the Hidex Sense 425-301 reader (Hidex, Turku, Finland). Quality of the assay was determined by Z-factor using cell-free wells as negative and wells containing DMSO treated cells as positive controls. Data were normalized to DMSO treated controls and curve fitting was performed to determine EC_50_ values for cell viability using a sigmoidal, 4PL model in GraphPadPrism (San Diego, CA, USA).

MTT assay. Vero cells were seeded into 96-well plates and allowed to attach for 24 h. Culture medium was removed and replaced with medium containing DMSO or compounds at desired concentrations. Cells were incubated for 48 h. Medium was removed and replaced with 100 μL DMEM containing 15 μL MTT ((3-(4,5-dimethylthiazol-2-yl)-2,5-diphenyl tetrazolium bromide)) (Sigma-Aldrich, St. Louis, MO, USA) per well and incubated for 2 h until crystals were present. MTT solution was removed from the cells, 100 μL DMSO was added to dissolve the crystals and optimal density OD was measured at 540 nm after 15 min.

### 2.12. siRNA-Mediated Depletion of OGG1

Cells were reverse-transfected with individual or with a pool of 4 siRNAs (Dharmacon, Lafayette, CO, USA) specific for OGG1 or with positive control AllStars Hs Cell Death Control siRNA (Qiagen, Hilden, Germany) or negative control ON-TARGETplus Non-targeting Pool siRNAs (Dharmacon, Lafayette, CO, USA). siRNAs were dissolved in 30 μL serum-free DMEM containing 0.6 μL INTERFERin transfection reagent (PolyPlus Transfection, Illkirch-Graffenstaden, France) per well, mixed by pipetting 10 times and incubated at room temperature for 10–15 min for transfection complex formation. U2-OS cells were seeded on top of transfection complexes in 70 μL complete DMEM medium with final density 3000 cells per well, yielding final siRNA concentration 10 nM. Cells were incubated in the presence of siRNA transfection for 48 h. Details of siRNAs used are found in the Table S1.

### 2.13. Cellular Thermal Shift Assay (CETSA)

Cetsa-TR. CETSA was performed with intact cells as described previously [27]. Briefly, U87 or SW13 cells were seeded on 2 × T75 flasks. The following day, cells were incubated with DMSO (0.2% *v*/*v*) or 20 μM TH6744 for 1.5 or 4 h at 37 °C. Cells were then washed with PBS containing DMSO or compound, harvested by TryplE Express (Gibco, USA) and resuspended in TBS supplemented with cOmplete™, Mini, EDTA-free Protease Inhibitor Cocktail (Roche, Basel, Switzerland) at 1.0 × 10^5^ cells per 100 μL. Cells from each condition were divided to 10 aliquots and heated to different temperatures (37, 41, 44, 47, 50, 53, 56, 59, 63 or 67 °C) for 3 min using Veriti 96-well Thermal Cycler (Applied Biosystems, Foster City, CA, USA) followed by cooling at 25 °C for 3 min. Thereafter, the cells were lysed by 3 freeze–thaw cycles using dry ice in 100% Ethanol and a water bath set at 25 °C. The lysates were then clarified by centrifugation at 20,000× *g* for 40 min at 4 °C and prepared for Western blotting to detect Hsp70 or OGG1. SOD-1 served as the loading control.

Isothermal dose response Cetsa. U87 or SW13 (2 × 10^5^) cells were seeded on a 6-well plate. The following day, cells were incubated with DMSO (0.3% *v*/*v*) or different TH6744 concentrations for 1.5 or 4 h at 37 °C. Cells were washed with PBS containing DMSO or compound at respective dose, harvested by TryplE Express and resuspended in 100 μL PBS supplemented with DMSO or compound at respective dose. Cells were heated at 47 °C (for detecting OGG1), 56 °C (Hsp70) or 59 °C (Hsp70) for 3 min using Veriti 96-well Thermal Cycler (Applied Biosystems, Foster City, CA, USA) followed by cooling at 25 °C for 3 min. Thereafter, the cells were lysed by 3 freeze–thaw cycles using dry ice in 100% Ethanol and a water bath set at 25 °C. The lysates were clarified by centrifugation at 20,000× *g* for 40 min at 4 °C and prepared for Western blotting to detect Hsp70 or OGG1. SOD-1 or β-actin served as the loading control.

### 2.14. Western Blot

Upon sample preparation and clarification by centrifugation, lysates containing 20–30 μg total protein (measured using Pierce™ BCA Protein Assay Kit, Thermo Fisher Scientific, Waltham, MA, USA) were mixed with 4× Laemmli buffer (Bio-Rad, Hercules, CA, USA) supplemented with mercaptoethanol (Sigma-Aldrich, St. Louis, MO, USA) or Dithiothreitol (DTT) (Sigma-Aldrich, St. Louis, MO, USA). Samples were heated at 95 °C for 10 min. Proteins were separated in 4–15% Mini-PROTEAN TGX gels (Bio-Rad, Hercules, CA, USA) and transferred to nitrocellulose membranes using a Trans-Blot Turbo machine (Bio-Rad, Hercules, CA, USA). Membranes were blocked in Odyssey Blocking Buffer (LI-COR, Lincoln, NE, USA) or in 2% non-fat milk (Sigma-Aldrich, St. Louis, MO, USA) in TBS-T, incubated with primary antibodies (details of antibodies is found in Table S2) against desired protein at 4 °C overnight and with fluorescence-conjugated species-specific secondary antibodies at room-temperature for 1 h. Membranes were washed three times with TBS-T between incubations. Protein bands were directly visualized with an Odyssey Fc Imager (LI-COR, Lincoln, NE, USA) and images were analysed using Image Studio Lite Software (LI-COR, Lincoln, NE, USA). All raw uncropped images are compiled in Data S7.

### 2.15. In Vitro OGG1 Activity Assay

Inhibition of human OGG1 enzymatic activity was measured as described previously [23]. Briefly, compounds were mixed with 0.8 nM OGG1 enzyme, 2 nM AP endonuclease 1, and 10 nM oligonucleotide duplex DNA (5ʹ-FAM-TCTGCCA[8-oxoA]CACTGCGTCGACCTG-3ʹ, annealed to 5ʹ-CAGGTCGACGCAGTGCTGGCAGT[Dabcyl]-3ʹ, where [FAM] indicates fluorescein, and [8-oxoA] indicates 8-oxo-2ʹ-deoxyadenosine (TriLink Biotech, San Diego, CA, USA) in a buffer containing 25 mM Tris-HCl pH 8.0, 15 mM NaCl, 2 mM MgCl2, 0.5 mM DTT, and 0.0025% Tween-20. Fluorescein fluorescence was then recorded in a Hidex Sense plate reader.

### 2.16. Thermal Proteome Profiling Sample Preparation

TPP-TR. SW-13 cells were seeded into T175 flasks, after 24 h infected with HAZV (MOI 10) for 16 h and treated with 20 μM TH6744 or DMSO control (0.2% *v*/*v*) for 4 h. Cells were washed twice with PBS, harvested by TryplE Express (Gibco, USA) and collected in PBS. Cells were centrifuged at 300× *g* for 5 min, re-suspended in 1100 μL PBS containing the compound or DMSO, divided in ten 100 μL aliquots and heated individually for 3 min at 37, 41, 44, 47, 50, 53, 56, 59, 63, and 67 °C using Thermocycler (Applied Biosystems, Foster City, CA, USA) followed by cooling at 25 °C for 3 min. Thereafter, the cells were freeze–thawed 3 times by using dry ice in 100% Ethanol and a water bath set at 25 °C. Precipitated proteins were separated from soluble proteins by centrifugation at 20,000× *g* for 40 min at 4 °C. In total, 70 μL supernatants were transferred into new microtubes and supplemented with SDS to reach final concentration 0.1%. Total protein concentration was measured by PierceTM BCA Protein Assay Kit (Thermo Fisher Scientific, Waltham, MA, USA). TPP-TR experiments were conducted as two biological replicates.

2D-TPP. U87 cells were seeded into T175 flasks 72 h prior assay to allow cell growth and obtain approximately 80% confluency. Cells were treated with 30, 10, 3, and 1 μM TH6744 or DMSO for 1.5 h. Cells were washed twice with PBS containing corresponding compound dose, harvested by TryplE Express (Gibco, USA) and collected in PBS containing compound. Cells were centrifuged at 300× *g* for 3 min, re-suspended in 1000 μL PBS containing the compound or DMSO. Cells from each compound treatment were divided in 9 × 100 μL aliquots and heated individually for 3 min at 42, 45, 48, 51, 54, 57, 60, 63, and 67 °C using Thermocycler (Applied Biosystems, Foster City, CA, USA) followed by cooling at 25 °C for 3 min. Thereafter, the cells were freeze–thawed 3 times by using dry ice in 100% Ethanol and a water bath set at 25 °C. Precipitated proteins were separated from soluble proteins by centrifugation at 20,000× *g* for 30 min at 4 °C. In total, 70 μL supernatants were transferred into new microtubes. Total protein concentration was measured by PierceTM 660 nm Protein Assay Kit (Thermo Fisher Scientific, Waltham, MA, USA).

### 2.17. Sample Preparation for LC-MS/MS

Protein digestion and peptide labelling. Total protein concentration of samples was determined by using a DC™ Protein Assay (Bio-Rad, Hercules, CA, USA). Supernatants were supplemented with a stock solution of sodium dodecyl sulfate (SDS) (Sigma-Aldrich, St. Louis, MO, USA), tris(2-carboxyethyl)phosphine (TCEP) (Sigma-Aldrich, St. Louis, MO, USA) and triethylammonium bicarbonate (TEAB) (Sigma-Aldrich, St. Louis, MO, USA) to reach the final concentrations of 0.1% SDS, 5 mM TCEP and 50 mM TEAB. Samples were reduced for 30 min at 65 °C and subsequently alkylated by chloroacetamide at a final concentration of 15 mM for 30 min at RT. At a first digestion step, the protease LysC (Wako, Osaka, Japan) was added to each sample at a protease-to-protein ratio of 1:55 (for TPP-TR) or 1:40 (for 2D-TPP) with regard to the protein amount in the sample with the highest total protein concentration. Digestion was allowed to take place at 37 °C for 16 h. Proteins were digested by adding Pierce™ Trypsin Protease, MS Grade (Thermo Fisher Scientific, Waltham, MA, USA) at a ratio of 1:60 (TPP-TR) or 1:70 (2D-TPP) and incubation for 8 h, followed by adding trypsin once more at a ratio of 1:60 (TPP-TR) or 1:70 (2D-TPP) to complete digestion during a second overnight incubation at 37 °C. Samples were labeled with isobaric TMT tags (Thermo Fisher Scientific, Waltham, MA, USA) according to the manufacturer’s instructions, but adjusted for three hours. In TPP-TR the full temperature range of each condition (ten samples) was labeled by the ten TMT tags of one TMT10-plex. In 2D-TPP three TMTpro-sets were used, excluding the 134N channel. Each set included samples from five different compound concentrations (0, 1, 3, 10, and 30 μM) and were distributed in a way that the first set comprised the samples that were melted at 42, 45 and 48 °C, the second one at 51, 54 and 57 °C and the third at 60, 63 and 67 °C. Labelling efficiency was determined by LC-MS/MS before pooling of samples into 10- or 15-plexes for TPP-TR and 2D-TPP experiments, respectively. Sample clean-up was performed by solid phase extraction (SPE strata-X-C, Phenomenex, Torrance, CA, USA) and purified samples were dried in a SpeedVac (Thermo Fisher Scientific, Waltham, MA, USA).

Prefractionation by high pH reversed-phase chromatography (HpH RP-HPLC). In the TPP-TR experiment, sample pools were pre fractionated using HpH RP-HPLC [28] and 200 μg of peptide mixture from each set were fractionated using a Waters XBridge BEH300 C18 3.5 μm 2.1 × 250 mm column (Waters Corporation, Milford, MA, USA) on a Agilent 1200 HPLC system operating at 200 μL per min. Buffer A consisted of 20 mM NH3, and buffer B of 80% acetonitrile, 20 mM NH3. Fractions were eluted using a gradient from 3 to 88% B in 63 min. The column was washed with 88% B for 15 min, followed by a ramp to 100% B in 2.5 min and kept at 100% B for another 13.5 min. Fractions were collected into a polypropylene V-96-well microtiter plates (Microplate, 96-well PP, V-Bottom; Grainer BIOONE, Kremsmünster, Austria). At 97 min, fraction collection was halted, and the gradient was held at 3% B for 20 min. The total number of concatenated fractions was set to 12. Each plate was dried at RT using a SpeedVac (SPD 111V, Thermo Fisher Scientific, Waltham, MA, USA). Plates were stored at −20 °C until LC/MS/MS analysis.

Prefractionation by high-resolution isoelectric focusing (HiRIEF). In the 2D-TPP experiment, HiRIEF prefractionation method was applied as previously described [29]. Briefly, 300 μg of the dried peptide sample pools were subjected to peptide IEF-IPG (isoelectric focusing by immobilized pH gradient) in the pI range of 3–10. Peptide samples were dissolved in a 250 μL rehydration solution containing 8 M urea and 1% IPG pharmalyte pH 3–10 (GE Healthcare, Chicago, IL, USA) and allowed to adsorb to the gel strip by swelling overnight. The peptides were focused on the gel strip and were thereafter passively eluted into 72 contiguous fractions into a V-bottom 96-well plate (Grainer BIOONE, Kremsmünster, Austria; product #651201) with MilliQ water/35% ACN/35% ACN + 0.1%FA using an in-house built IPG extractor robotics system (GE Healthcare, Chicago, IL, USA, prototype instrument). Samples were dried by using a SpeedVac (Thermo Fisher Scientific, Waltham, MA, USA) at RT and kept at −20 °C. 

### 2.18. Liquid Chromatography Tandem Mass Spectrometry (LC-MS/MS) Analyses

LC-MS/MS runs of the HpH RP-HPLC fractions (TPP-TR). Each sample was analyzed on a HF Q-Exactive Orbitrap (Thermo Fisher Scientific, Waltham, MA, USA) connected to a Dionex UHPLC system (Thermo Fisher Scientific, Waltham, MA, USA). The UHPLC was equipped with a trap column Acclaim PepMap 100, 75 μm × 2 cm, nanoviper, C18, 3 μm, 100 Å (Thermo Fisher Scientific, Waltham, MA, USA) and an analytical column PepMap RSLC C18, 2 μm, 100 Å, 75 μm × 50 cm (Thermo Fisher Scientific, Waltham, MA, USA). Mobile-phase buffers for nLC separation consisted of 0.1% FA in water (solvent A) and 80% acetonitrile/0.1% formic acid (solvent B). The peptides were eluted during a 2 h gradient and directly sprayed into the mass spectrometer. The flow rate was set at 250 nL per min, and the LC gradient was as follows: 3–6% solvent B within 3 min, 6–35% solvent B within 117 min, 35–47% solvent B within 5 min, 47–100% solvent B within 5 min and 100% B for 8 min and 1% solvent B for 5 min. The mass spectrometer was operated in a data-dependent acquisition mode (top 10 most intense peaks) performing a survey scan from 370 to 1600 m/z (resolution 60,000). Automatic gain control (AGC) target was set to 3e6 and maximum injection time to 250 ms. MS2 scans were acquired on the 10 most-abundant MS1 ions of charge state 2–7 using a Quadrupole isolation window of 1 m/z for higher-energy collision dissociation HCD fragmentation. Collision energy was set to 34%. MS2 spectra were acquired at a resolution of 30,000 using an AGC target of 2e5 and a maximum injection time of 200 ms. Dynamic exclusion was set to 15 s. The mass spectrometry proteomics data have been deposited to the ProteomeXchange Consortium via the PRIDE [30] partner repository with the dataset identifier PXD021494.

LC-MS/MS runs of the HiRIEF fractions (2D-TPP). Online LC-MS was performed as previously described [29] using a Dionex UltiMate™ 3000 RSLCnano System (Thermo Fisher Scientific, Waltham, MA, USA) coupled to a Q-Exactive-HF mass spectrometer (Thermo Fisher Scientific, Waltham, MA, USA). Peptides of each of the 72 plate wells were dissolved in 0.1% formic acid, 3% acetonitrile in water and subjected to LC-MS/MS analysis. Samples were trapped on a C18 guard-desalting column (Acclaim PepMap 100, 75 μm × 2 cm, nanoViper, C18, 5 μm, 100 Å; Thermo Fisher Scientific, Waltham, MA, USA), and separated on a 50 cm long C18 column (Easy spray PepMap RSLC, C18, 2 μm, 100 Å, 75 μm × 50 cm; Thermo Fisher Scientific, Waltham, MA, USA). The nano capillary solvent A consisted of 95% water, 5% DMSO and 0.1% formic acid. Solvent B consisted of 5% water, 5% DMSO, 95% acetonitrile and 0.1% formic acid. At a constant flow of 0.25 μL min−1, the curved gradient went from 10% B up to 40% B for fraction 1–19, 8% B up to 40% B for fraction 20–39 and 6% B up to 40% B for fraction 40–72 in a dynamic range of gradient length, followed by a steep increase to 100% B in 5 min. FTMS master scans with 60,000 resolution (and mass range 300–1500 m/z) were followed by data-dependent MS/MS (30,000 resolution) on the top five ions using higher energy collision dissociation (HCD) at 30% normalized collision energy. Precursors were isolated with a 2 m/z window. Automatic gain control (AGC) targets were 1E6 for MS1 and 1E5 for MS2. Maximum injection times were 100 ms for MS1 and 100 ms for MS2. Dynamic exclusion was set to 30 s duration. Precursors with unassigned charge state or charge state 1 were excluded. An underfill ratio of 1% was used.

### 2.19. Peptide and Protein Identification and Quantification

The raw data for the TPP-TR experiment were analyzed as previously described [31]. Mascot was used to search the MS/MS data against the UniProt Homo sapiens database (UniProt Consortium) (containing canonical and isoforms_42144 entries downloaded on 21st of March 2016).

The raw data for the 2D-TPP experiment were analyzed as previously described [32]. Mascot was used to search the MS/MS data against the UniProt Homo sapiens database (UniProt Consortium) (containing canonical and isoforms_42144 entries downloaded on 21st of March 2016).

### 2.20. TPP Statistical Analysis and Hit Selection

TPP-TR: TPP-TR results were analyzed as described previously [31,33]. First, proteins were sorted by ascending non-parametric analysis of the response curve (NPARC) *p*-value “p_adj_NPARC”. The adjusted *p*-value from a non-parametric statistical test of the curve-fitting step was used to generate the data in the TPP R package. A 0.0001 threshold was applied, yielding protein melt curves with significant ΔTm (1589 proteins). Next, proteins with a “p_adj_NPARC” below threshold were filtered by the column “meltP_diffs_have_same_sign”, selecting rows that contain a “yes” meaning that the ΔTm between DMSO_1 and TH6744_1 has the same sign (positive or negative) as the ΔTm between DMSO_2 and TH6744_2 (1224 proteins). Next, proteins were filtered on the column “meltP_diffs_T_vs_V_greater_V1_vs_V2”, selecting rows that contain a “yes”, meaning that the ΔTm between treatment and DMSO is larger than between DMSO_1 and DMSO_2 replicates (1154 proteins). Last, a ΔTm > 2 °C threshold was applied (593 proteins).

2D-TPP: 2D-TPP results were analyzed as described previously [33,34]. Proteins with changed solubility or expression were excluded and F-statistic threshold of 15 was applied. Half-maximal effective concentration of protein stabilization/destabilization pEC_50_ (pEC_50_ = −log(EC_50_) illustrates binding affinity of TH6744 to proteins.

### 2.21. Gene Set Enrichment and Interaction Analyses

All gene set enrichment analyses were performed using Metascape (http://metascape.org/gp/index.html#/main/step1). Gene annotation terms for enrichment analysis were extracted from CORUM, Reactome gene sets, KEGG pathways, gene ontology molecular function and gene ontology biological process databases. Enriched terms with *p* < 0.01, 3 or more genes and a ratio between observed and expected count >1.5 were clustered by hierarchical clustering based on term similarities and using kappa scores as a similarity metric. Terms were selected for representation of a cluster when they had the highest statistical significance of the cluster. Protein–protein interaction networks were extracted from the STRING database (STRING Consortium) with a 0.7 confidence threshold and excluding interactions extracted from text mining.

### 2.22. Statistical Analysis

Statistical significance was determined using the GraphPad Prism Software (San Diego, CA, USA, Version 8.4.0). Two-tailed *t*-tests, one- or two-way ANOVA with appropriate follow up tests were used. For each data panel, the tests used are stated in the according figure legend. If not indicated otherwise, data are presented as mean ± SD from at least *n* = 2 biological replicates. Threshold for defining significance was *p* < 0.05 and significance levels were defined as follows: * *p* < 0.05, ** *p* < 0.01, *** *p* < 0.001. For dose-response-testing curve fitting was performed to determine IC_50_ using a sigmoidal, 4PL model in GraphPad Prism (San Diego, CA, USA) with bottom and top constraints at 0 and 100, respectively. If not indicated otherwise, each image shown is a representative of at least *n* = 2 biological replicates.

### 2.23. Data Availability

The data that support the findings of this study are within the article, Supplementary Materials, supplementary data or available from the corresponding author upon request. Data S3 is accessible in 10.17044/scilifelab.13089023. The raw mass spectrometry data have been deposited to the ProteomeXchange Consortium via the PRIDE partner repository with the dataset identifier PXD021494.

## 3. Results

### 3.1. High-Throughput Phenotypic Screening on HAZV-Infected Cells Identifies TH3289 and TH6744 as Antiviral Compounds

**Figure 1:**
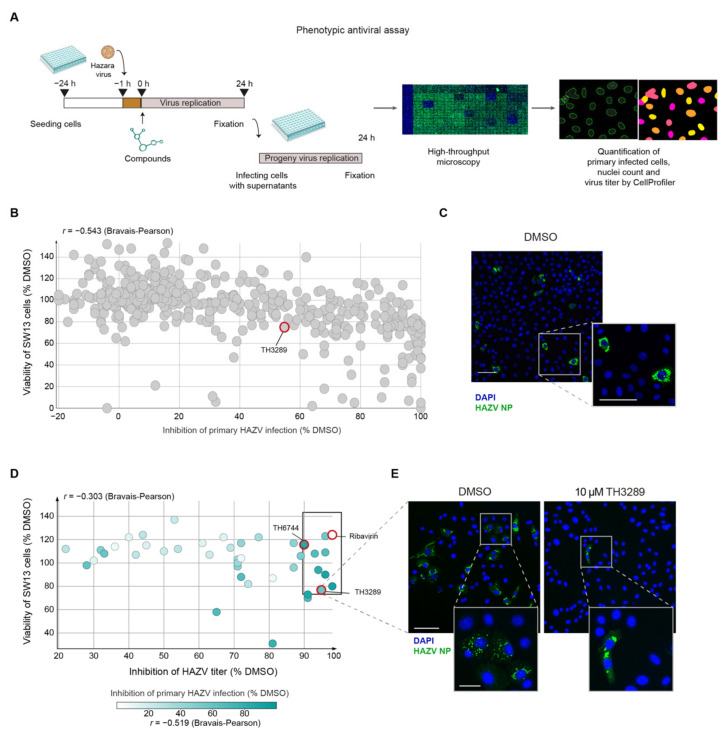
Discovery of several novel antiviral compounds by an image-based phenotypic Hazara virus (HAZV) screen. (**A**) Schematic overview of phenotypic antiviral screening cascade of small molecule compounds, evaluating nuclei count as an indicator of compound toxicity, level of HAZV infection in cells by HAZV nucleoprotein (NP) antibody staining (primary infection) and HAZV progeny release by end-point dilution assay (virus titer). Automated image analysis was performed. (**B**–**E**), SW13 cells were infected with HAZV (MOI 10) and treated with 10 μM of compounds from the in-house library for 24 h. Cells were stained for DAPI (blue) and HAZV NP (green) and analyzed by high-throughput microscopy. Data are presented as a mean of two technical replicates per compound performed in *n* = 1 biological replicate. (**B**) In primary infection screen, % inhibition of HAZV infection was determined based on HAZV NP and DAPI signal relative to DMSO. Viability of SW13 cells was based on nuclei count relative to DMSO. (**C**) Representative images of HAZV infected SW13 cells. DAPI in blue and HAZV NP in green. Scale bars equal 100 μm. (**D**) Virus titer from supernatant was determined by end-point dilution assay on Vero cells and quantified by automated image analysis. Viability of SW13 cells was based on nuclei count relative to DMSO from the primary infection screen. (**E**) A representative image of HAZV infected Vero cells treated with DMSO or 10 µM TH3289. DAPI in blue and HAZV NP in green. The upper image scale bar equals 100 μm, the lower image scale bar equals 20 μm.

To test the antiviral activity of our in-house small molecule inhibitor library targeting cell oxidative stress and nucleotide metabolism pathways against emerging RNA viruses, a phenotypic antiviral assay was established (Figure 1A). Since screening for antivirals in high-containment biosafety level (BSL) settings is challenging, human non-pathogenic Hazara virus (HAZV), that belongs to the same serogroup of CCHFV [35] but can be handled in a BSL2 laboratory, was chosen. Adrenal gland carcinoma SW13 cells were infected with HAZV, followed by compound treatments. To assess antiviral activity and cytotoxicity of tested compounds, percentage of HAZV-infected cells and nuclei count were quantified by high-throughput microscopy and automated image analysis using CellProfiler software (Broad Institute, Cambridge, MA, USA) (Figure 1A). Additionally, newly produced viral particles were quantified by re-infecting Vero cells with supernatants from primary infected cells (Figure 1A). The quality of antiviral assays was confirmed by Z-factors [26] of 0.83 for primary and 0.4 for viral titer (Figure S1). In total, 425 compounds from the in-house library targeting oxidative stress and nucleotide metabolism were tested at a single dose of 10 µM for 24 h in four separate screening rounds (Figure 1B). In total from all screened compounds, 14 compounds inhibited primary virus infection by 90% alongside cell viability higher than 80% (Figure 1B,C; Data S1), while the majority of compounds had limited effects on HAZV replication and on cell viability. To find compounds affecting production of new virus particles, few compounds from each primary screening round showing varying activity on primary infection were chosen for a follow-up study determining effects on HAZV viral titer. Eight compounds from the in-house library reduced the viral titer by 1-log-unit (Figure 1D; rectangle). Clinically used antiviral Ribavirin at a dose of 100 µM served as a positive control in the assay reducing HAZV viral titer by 98% (Figure 1D). Compound TH3289 was a hit from the first titer screening, while TH6744 was identified during the second screening inhibiting HAZV titer by 95% and 90%, respectively (Figure 1D,E) and were chosen for follow-up studies. Correlation scores of −0.543 and −0.303 between cell viability and inhibition of primary infection or viral titer inhibition, respectively, showed no correlation (Figure 1B,D), indicating that the antiviral effect was not caused by cell toxicity. Taken together, new small-molecule antivirals TH3289 and TH6744 were identified using an image-based high-throughput screening cascade quantifying virus infected cells on a single-cell resolution in an automated manner.

### 3.2. Compound TH3289 Has Broad Antiviral Activity Spectrum among Emerging RNA Viruses Including SARS-CoV-2, EBOV and CCHFV

**Figure 2:**
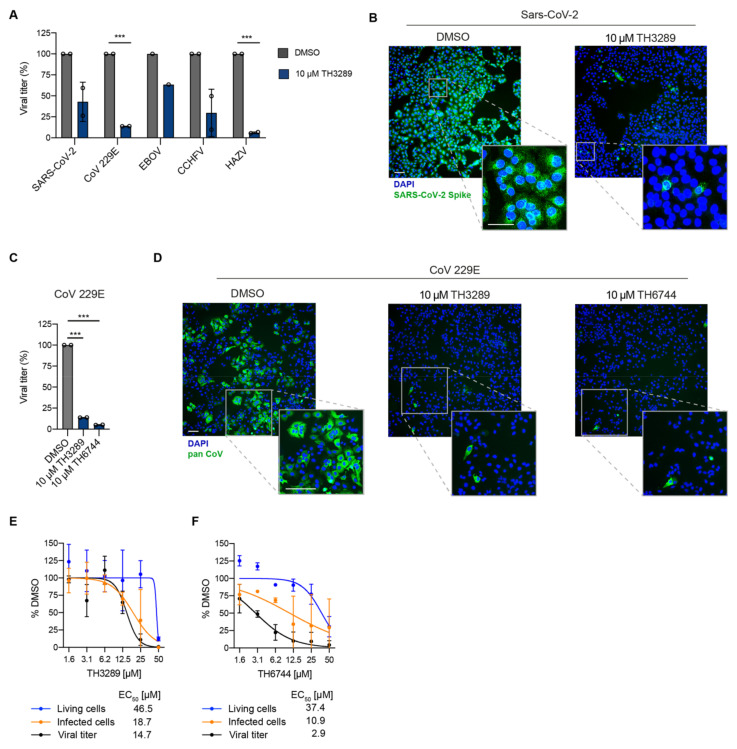
Validation of broad-spectrum activity and wide therapeutic window of the novel antiviral compounds. (**A**) Vero E6 cells were infected with SARS-CoV-2 (MOI 0.05), Huh7 cells were infected with CoV 229E (MOI 5), Vero cells were infected with EBOV (MOI 0.5), CCHFV (MOI 1) and SW13 cells were infected with HAZV (MOI 1). Infection was followed by treatment with 10 μM TH3289 for 24 h (SARS-CoV-2, CoV 229E, HAZV) or 48 h (EBOV, CCHFV) and end-point dilution assay was performed on Vero E6 (SARS-CoV-2), Vero cells (CCHFV, EBOV) or Huh7 cells (CoV 229E). Data are presented as mean ± SD from *n* = 2 (SARS-CoV-2, CoV 229E, CCHFV, HAZV) and *n =* 1 (EBOV) biological replicates. Statistical significance was determined using unpaired two-tailed *t*-tests. *** *p* < 0.001. (**B**) Representative images from Vero E6 cells infected with SARS-CoV-2 (MOI 0.05) and treated with 10 μM TH3289 or DMSO. Cells were stained for SARS-CoV-2 Spike (green) and DAPI (blue). Scale bars equal 100 μm. (**C**) Huh7 cells were infected with CoV 229E (MOI 5). Infection was followed by treatment with 10 μM TH3289 or 10 μM TH6744 for 24 h and end-point dilution assay was performed on Huh7 cells. Data are presented as mean ± SD from n = 2 biological replicates. Statistical significance was determined using unpaired two-tailed *t*-tests. *** *p* < 0.001. (**D**) Representative images from Huh7 cells infected with CoV 229E (MOI 5) and treated with 10 μM TH3289, 10 μM TH6744 or DMSO. Cells were stained for Pan Coronavirus (green) and DAPI (blue). Scale bars equal 100 μm. (**E**,**F**) SW13 cells infected with HAZV (MOI 1) and treated with indicated doses of TH3289 (**E**) or TH6744 (**F**). Cell viability was determined by nuclei count (blue line), infected cells by HAZV-NP staining (orange line) and virus titer by end-point dilution assay (black line). Data are presented as Mean ± SD from *n =* 2 biological replicates. Curve fitting was performed to determine IC_50_.

To study if the series of in-house compounds may exhibit antiviral activity beyond non-pathogenic HAZV, two of the screening hits, TH3289 and TH6744 activity were tested on human pathogenic viruses. Antiviral activity of TH3289 was assessed on RNA viruses SARS-CoV-2, CoV 229E, EBOV and CCHFV. Treatment with 10 µM TH3289 reduced SARS-CoV-2 titer by 58% in Vero E6 cells (Figure 2A,B) and CoV-229E titer by 84% in Huh7 cells at non-toxic concentrations (Figure 2A,C,D; Figure S2A). Furthermore, viral titers of EBOV and CCHFV infections were reduced by 40% and 70% in Vero cells at non-toxic concentrations, respectively (Figure 2A; Figure S2B). TH3289 reduced HAZV titer in SW13 cells by 94% (Figure 2A), validating the antiviral screening findings. Similar to TH3289 treatment, treatment with 10 µM TH6744 reduced CoV 229E titer by 95% (Figure 2C,D; Figure S2C). Taken together, these results highlight the relevance of our compound library, in particular TH3289 and TH6744 across virus families, especially showing antiviral activity against Coronaviruses, in cell lines susceptible for corresponding virus infections.

Further, to validate the antiviral screening hits and examine the therapeutic window of top hit compounds, the EC_50_ values for TH3289 and TH6744 were determined in a dose-response treatment in HAZV-infected SW13 cells. The antiviral effect was confirmed for TH3289 and TH6744 compounds with EC_50_ values of 18.7 µM and 10.9 µM on primary infection and 14.7 µM and 2.9 µM on viral titer, respectively (Figure 2E,F; Data S2). Altogether, a dose-dependent decrease of infected cells and viral titer at non-toxic TH3289 and TH6744 concentrations was observed. In summary, TH6744 has a wider therapeutic window, is more potent at inhibiting both HAZV primary infection and progeny release and was thereby selected for further studies.

### 3.3. Antiviral Activity is Independent of OGG1 Inhibition

**Figure 3:**
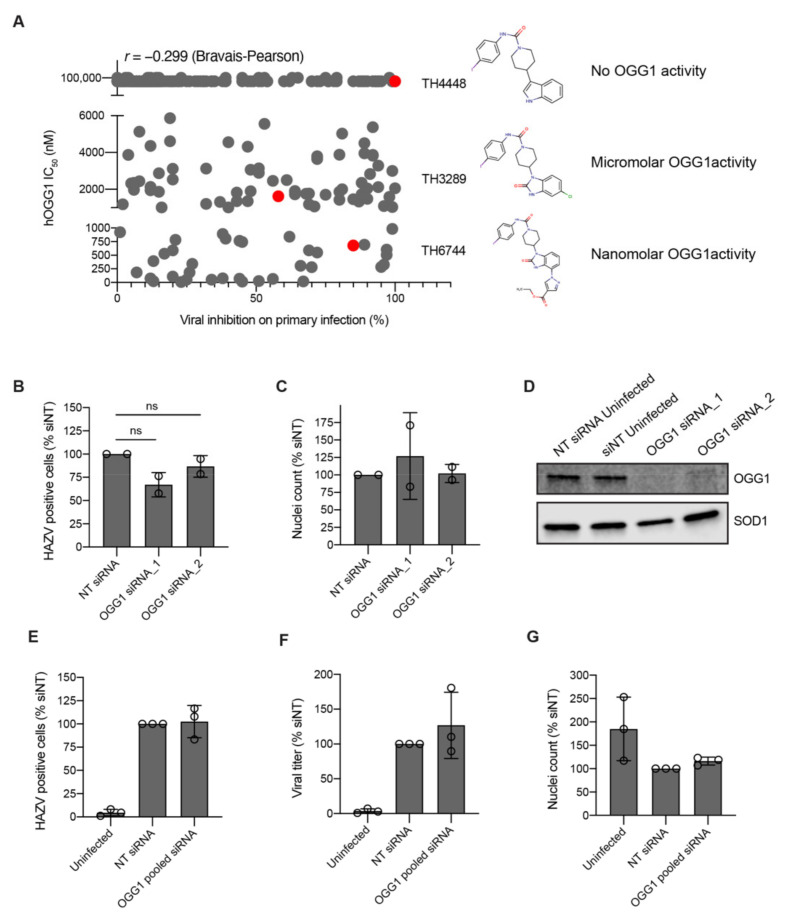
Antiviral activity is independent of OGG1 inhibition. (**A**) Correlation between compounds’ inhibition of HAZV primary infection and OGG1 activity. Antiviral activity was measured on primary infection in the HAZV phenotypic antiviral assay and IC_50_ of hOGG1-inhibition was determined by an in vitro OGG1 activity assay. Correlations were determined using the Bravais–Pearson correlation coefficient. Chemical structures of TH4448, TH3289 and TH6744. (**B**–**D**) U2OS cells were reverse transfected with two individual siRNAs targeting OGG1 mRNA for 48 h, followed by infection with HAZV for 24 h. Percentage of HAZV-positive cells (**B**) and nuclei count (**C**) at 24 hpi. Data are expressed as mean ± SD of *n =* 2 biological replicates and statistical significance was determined by using one-way ANOVA with Dunnett’s multiple comparison analysis. (**D**) Representative Western blot image of OGG1 protein levels upon OGG1 siRNA depletion after 72 h. (**E**–**G**) U2OS cells were reverse transfected with four pooled siRNAs targeting OGG1 for 48 h, followed by infection with HAZV (MOI 1) for 24 h. Percentage of HAZV-positive cells (**E**), titer in Vero cells (**F**) and cell viability (**G**) were quantified at 24 hpi. Data are expressed as mean ± SD of *n =* 3 biological replicates. Statistical significance was determined by using one-way ANOVA with Dunnett’s multiple comparison analysis.

In-house compound library that was used for antiviral screening is designed to target proteins involved in oxidative stress and nucleotide metabolism pathways. The two hit compounds TH3289 and TH6744 belong to a series designed to target human 8-oxoguanine glycosylase 1 (OGG1) [23]. We thereby studied the role of OGG1 on RNA viruses. Compounds screened in HAZV-infected cells (see Figure 1 and Data S1) were assessed by in vitro OGG1 activity assay. Interestingly, the inhibition of HAZV primary infection did not correlate with the compounds’ OGG1 IC_50_-values as depicted by low Bravais–Pearson correlation score of 0.299 (Figure 3A). As an example, IC_50_ of OGG1 inhibition for TH6744 and TH3289 are 680 nM and 1.6 μM, respectively, while analogue TH4448 showed no OGG1 inhibition (IC_50_ > 99 μM) (Figure 3A). Yet these three compounds show very similar levels of HAZV inhibition (Figure 3A), indicating that OGG1 inhibition does not solely explain antiviral phenotype of compounds. The structures of TH3289 and TH6744 are very similar: they are both benzimidazolones substituted with a piperidine ring carrying an aromatic urea moiety and carries either a small substituent on position 5 or a bulky substituent on position 4. The OGG1-inactive compound TH4448 carries a piperidine ring with an aromatic urea moiety, but lacks both the 2-oxo group and additional substituents on the benzene ring.

To further elucidate the role of OGG1 activity in virus infection, HAZV infection was studied upon OGG1 downregulation using small-interfering RNA (siRNA). HAZV replication or cell viability was not impaired upon OGG1 depletion in U2OS cells using individual siRNA sequences (Figure 3B–D) nor when using a pool of 4 different siRNA sequences simultaneously (Figure 3E–G). Thus, we conclude that the antiviral effect seen with our compounds is independent from their OGG1 inhibitory function.

### 3.4. Thermal Proteome Profiling Reveals Changes in Thermal Stability of Host Proteins upon TH6744 Treatment

**Figure 4:**
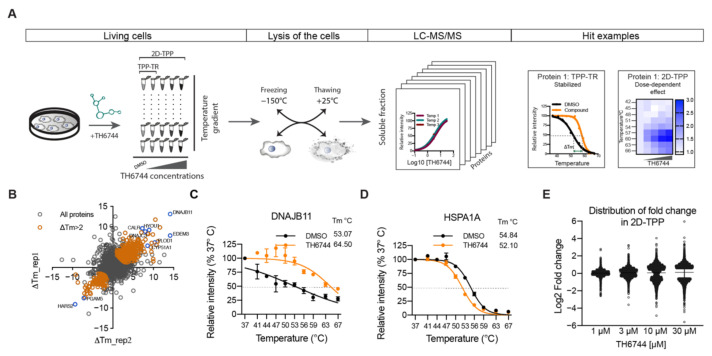
Effects of TH6744 on proteome thermal stability in living cells. (**A**) TPP-TR and 2D-TPP experimental workflow. (**B**) ΔTm across the proteome of HAZV-infected SW13 cells treated with 20 μM TH6744 in TPP-TR. Number of proteins were identified as gene symbol centric. Data are from one experiment, samples carried out in biological duplicate. Colored dots indicate proteins that passed selection criteria (see Methods) and in addition have ΔTm > 2 °C. (**C**,**D**) TPP-TR melt curves of DNAJB11 (**C**) and or HSPA1A (**D**). Normalized relative protein intensities from TPP-TR were fitted using sigmoidal 4-parameter curve fitting. Data are expressed as mean ± SD from *n =* 2 biological replicates. (**E**) Distribution of relative protein abundance as Log2 fold change after indicated TH6744 treatments compared to DMSO. Data are from the 2D-TPP experiment.

To study host target proteins and pathways that drive the antiviral activity, TH6744 was selected over TH3289 based on the wider therapeutic window on HAZV-infected cells. To determine target pathways of TH6744, protein–drug interactions were studied on a proteome-wide scale by applying two alternative TPP approaches. Temperature range TPP (TPP-TR) monitors protein–drug interactions over a temperature gradient upon treatment with a single compound dose measured by change in melting temperature (ΔTm) [18,31,36], while 2D-TPP determines drug potency to the target across increasing compound concentrations and temperatures (Figure 4A) [32]. Changes in the thermal stability of proteins may reflect direct binding of the compound to their target proteins or indicate either disturbance of protein complexes, subunits or indirect downstream effects [18]. TPP-TR was performed in HAZV-infected SW13 cells following 20 μM TH6744 treatment for 4 h. After confirming TH6744 binding to its known target OGG1 by Western blotting (Figure S3A) and TPP-TR sample quality control (Figure S3B), ΔTm of individual proteins were measured between DMSO and TH6744-treated samples (Figure S3C) [31]. TPP-TR analysis identified 447 stabilized and 146 destabilized proteins that passed selection criteria (see Methods) and in addition have ΔTm more than 2 °C (Figure 4B; colored data points) out of the total 7981 proteins identified (Figure 4B). In TPP-TR, ΔTm ranged between + 13.6 °C (DNAJB11; stabilized) (Figure 4C) and −9.2 °C (HARS2; destabilized) (Figure S3D). Enrichment analysis of these 593 proteins demonstrated mitochondrial matrix and protein folding as the most enriched GO terms (Figure S3E). For instance, regulators of protein folding pathways, DNAJB11 (Figure 4C), ER degradation-enhancing alpha-mannosidase-like protein 3 (EDEM3) (Figure S3F) and DNAJC3 (Figure S3G) were stabilized, while Hsp70 chaperone in cytosol (HSPA1A) was destabilized (Figure 4D). To narrow down target candidates, more comprehensive target identification by 2D-TPP was performed in human glioblastoma U87 cells, monitoring TH6744 effects dose-dependently at 1, 3, 10 and 30 μM treatment for 1.5 h over nine temperatures between 42–67 °C (Figure 4A). After confirming TH6744 binding to its known target OGG1 in U87 cells by Western blotting (Figure S3H) and 2D-TPP sample quality control (Figure S3I), dose-dependent thermal stability changes were measured. In the 2D-TPP analysis, a total of 8417 proteins were detected at 1% false discovery rate [37], where 220 proteins demonstrated dose-dependent thermal stability changes (94 stabilized and 126 destabilized) (Data S4 and Data S5). Higher compound concentrations induced thermal stability changes in larger fraction of proteins (Figure 4E). Majority of the 220 proteins were annotated as nucleotide-binding (98 proteins), ER-residents (41 proteins) or chaperones (16 proteins) (Uniprot annotation; Data S6). Collectively, the protein thermal stability changes indicate that TH6744 binds to a number of host proteins either directly or indirectly, thereby affecting multiple pathways.

### 3.5. Proteostasis Network is Enriched as Major TH6744 Target Pathway

**Figure 5:**
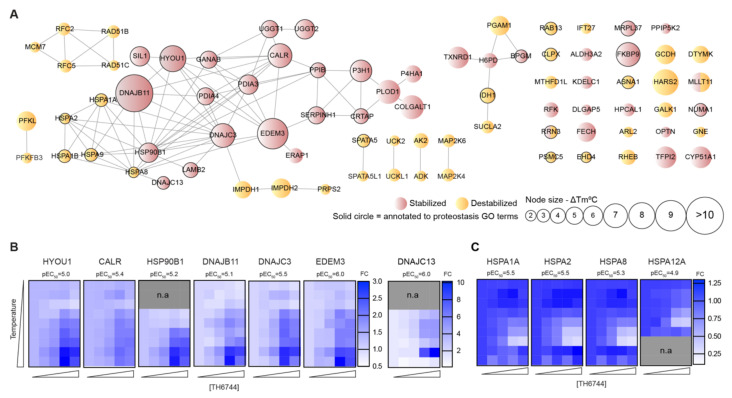
TH6744 treatment alters host proteostasis pathways. (**A**) Visualization of known and predicted protein–protein interaction network between 79 overlapping significant proteins in TPP-TR and 2D-TPP analysis by STRING database and Cytoscape tool with minimum required interaction score 0.7 (high confidence). Each node represents a protein, line represents an experimentally proven or predicted interaction, node size represents ΔTm between TH6744 and DMSO-treated sample from TPP-TR and node color represents stabilization (red) or destabilization (yellow) in TPP-TR and 2D-TPP analysis. Nodes with dual color had a differential effect on thermal stability between two assays. Nodes surrounded with black circles contribute to host proteostasis as annotated by GO terms. (**B**,**C**) Heat maps showing relative protein abundances as fold change of TH6744 treatment (*X*-axis, 1, 3, 10 and 30 μM) compared to DMSO treatment (first column in each graph) in presence of increasing temperatures (*Y*-axis, 42, 45, 48, 51, 54, 57, 60, 63 and 67 °C). Half-maximal effective concentration of protein stabilization/destabilization pEC_50_ (pEC_50_ = −log(EC_50_) illustrates binding affinity of TH6744 to host targets. *n =* 1 biological replicate. Grey squares indicate not available values. (**B**) Heat maps from 2D-TPP showing relative abundances of proteins typically located in the ER. (**C**) Heat maps from 2D-TPP showing relative abundances of proteins typically located in cytosol.

Next, overlapping hits from TPP-TR and 2D-TPP were further studied and 79 proteins had significant changes in both data sets, demonstrating >10% overlap from two independent TPP approaches (Figure S4A). Pathway analysis of overlapping hits revealed an enrichment of proteins involved in ER lumen, chaperone-mediated protein folding and ER unfolded protein response (Figure S4B) (22 proteins, e.g., DNAJC3, HSPA1A, HSPA8, HYOU1)—all involved in maintenance of proteostasis. Additionally, nucleoside monophosphate metabolic and biosynthetic processes were enriched (17 proteins, e.g., EDEM3 and UCK2). ER processes undergo heavy remodeling during infection of RNA viruses such as Coronaviruses [38] in order to accommodate the high demand of viral RNA and protein synthesis. Because of the strong enrichment of ER and protein homeostasis pathways in the TPP analyses, we decided to further study the effect of TH6744 on host proteostasis machinery. Protein–protein interaction analysis of the overlapping 79 proteins revealed a distinct interaction cluster formed by proteins involved in proteostasis (Figure 5A, solid circles). For instance, ER resident chaperones Hypoxia up-regulated protein 1 (HYOU1), Calreticulin (CALR), Endoplasmin (HSP90B1) and co-chaperones DNAJB11, DNAJC3, EDEM3 and DNAJC13 were stabilized (Figure 5B). Compound TH6744 binding affinity pEC_50_ score of 6 to EDEM3 and DNAJC13 illustrates strong binding at low-micromolar doses, while pEC_50_ of other co-chaperones is lower, illustrating weaker binding (Figure 5B). Contrary to ER-residents, chaperones typically located in cytosol (HSPA1A, HSPA2, HSPA8, HSPA12A) or mitochondria (HSPA9) are exclusively destabilized by TH6744 treatment (Figure 5C; Figure S4C). These data suggest a broad, subcellular location-dependent effect of TH6744 towards host factors maintaining proteostasis.

### 3.6. TH6744 Disturbs Interaction between Hsp70 and HAZV Nucleoprotein

**Figure 6:**
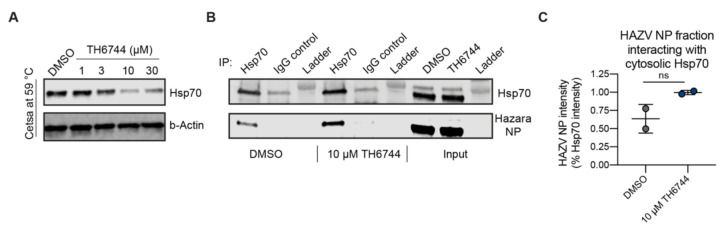
TH6744 increases the interaction between host cell Hsp70 and HAZV NP. (**A**) Isothermal TH6744 dose-response CETSA at 59 °C in living U87 cells. U87 cells were treated with indicated TH6744 doses for 1 h followed by CETSA at 59 °C and Western blotting. Data of *n =* 1 biological replicate are shown. (**B**) Immunoprecipitation of endogenous Hsp70 in HAZV-infected Hek293T cells. Hek293T cells were infected with HAZV (MOI 1) for 24 h, treated with 20 μM TH6744 for 1 h and processed for immunoprecipitation with Hsp70 antibody (detecting protein product of HSPA1A gene) or IgG control. Data are representative of *n* = 2 biological replicates. (**C**) Quantification of HAZV NP signal from B, HAZV NP signal is presented as relative to Hsp70 signal. Data are expressed as mean ± SD from *n =* 2 biological replicates. Statistical significance was determined by using unpaired Student’s *t*-test.

Viral protein synthesis and stability is dependent on host chaperones, especially widely noted is the involvement of Hsp70 in multiple viral replication cycle steps across virus families [39]. Based on this, we chose to investigate the effects of TH6744 treatment on one of the main proteostasis regulators HSP70 (encoded by HSPA1A gene). First, to validate TPP findings, Hsp70 thermal stability was evaluated by CETSA assays. Hsp70 destabilization was observed at 56 and 59 °C after TH6744 treatment measured by CETSA-TR in U87 (Figure S5A) and SW13 cells (Figure S5B) as well as dose dependently in U87 (Figure 6A) and SW13 cell lines (Figure S5C), validating TPP-TR and 2D-TPP findings, respectively. Next, to study the protein–protein interaction between Hsp70 and viral proteins upon TH6744 treatment, Hsp70 immunoprecipitation (IP) was performed in HAZV-infected cells. HAZV nucleoprotein (NP) was detected from Hsp70 pulldown fraction, but not from IgG control (Figure 6B), illustrating an interaction between Hsp70 and HAZV NP. Additionally, HAZV NP interaction with Hsp70 increased 2-fold (statistically not significant) upon TH6744 treatment measured by signal intensity (Figure 6B,C). Conclusively, these data show that TH6744 reduces Hsp70 thermal stability and alters the interaction between HAZV proteins and Hsp70.

## 4. Discussion

The increasing incidence of virus emergence and outbreaks in the last decade highlights the need for new, broadly acting antivirals, to diminish the impact of these diseases on health and economy. Phenotypic screening is an attractive approach to discover new host–pathogen interactions as well as new drug targets with potentially broad-spectrum activity [40]. Previously published antiviral screening cascades are often based on cell viability measured on primary infection [41,42] or replicon systems incapable of producing viable virus progeny [43]. Establishing an image-based phenotypic screening assay using infectious virus in biosafety laboratories allowed us to monitor all steps of the infection cycle. Additionally, follow-up study on virus titer enabled us to distinguish antiviral compounds that poorly inhibited infection in already infected cells, but significantly reduced viral progeny release and thereby virus transmission. Moreover, the developed image-based phenotypic assays provide a single-cell resolution and a detailed overview of the entire heterogeneous cell population instead of pooled population averages when using cell viability-based readouts. Image-based screenings have possibilities to uncover compound mechanistic insights when expanded to multidimensional phenotypic profiling [44].

Using the newly developed antiviral assay we first identified a small molecule inhibitor TH3289 as one of the top hit inhibiting HAZV progeny release. Upon the second screening round, a close analogue of TH3289 TH6744 was identified. Both TH3289 and TH6744 reduced HAZV titer to similar effect at 10-fold lower dose as marketed antiviral drug Ribavirin that has been previously shown to have in vitro activity against SARS-CoV-2 [45] and CCHFV [46] as well as in a clinical trial setting in combination with other antivirals in COVID-19 patients [47]. Interestingly, TH3289 had antiviral activity against all tested viruses, SARS-CoV-2, EBOV, CCHFV and CoV 229E, confirming its broad antiviral activity. TH3289 exhibited higher activity against CoV 229E, HAZV and CCHFV than EBOV and SARS-CoV-2, probably due to the differential roles of TH3289 target pathways in virus families. Given that testing compound activity in BSL3 and BSL4 laboratories were limited to the initial screening hit TH3289, the activity of TH6744 was validated in BSL2 setting on CoV 229E and had a significant antiviral activity. Further, high-throughput screenings using single compound doses require a validation of hit compounds. To validate the antiviral effect of our compounds, a dose-dependent activity of TH6744 and TH3289 was evaluated on HAZV primary infection, HAZV titer and cell nuclei. TH6744 exhibited better antiviral potency at non-toxic concentrations compared to TH3289 in a dose-response assay and was therefore chosen for follow-up target identification studies.

A challenge of phenotypic screening approaches is the lack of information about the target of identified compounds. TH6744 was developed as a part of a compound series targeting OGG1 and considering the link between oxidative stress and virus infection [19,20,21], we investigated the role of OGG1 inhibition on compounds’ antiviral properties. Surprisingly, we could not confirm OGG1 inhibition as the driver for the antiviral effect, pointing towards a multifactorial mechanism of action. It is widely appreciated that small molecule drugs often perform by poly-pharmacological activity [48]. Advanced proteomic techniques have recently been developed to study the effects of drug responses and to reveal compounds’ direct target and off-targets [32,49]. Additionally, TPP method has been used to study changes in cellular pathways in response to infection [50]. We implemented two distinct TPPs in living cells upon TH6744 treatment, shedding light on compound’s targets, secondary responses and effects on protein pathways. TPP-TR identified a plethora of host proteins responding to TH6744 treatment and therefore we applied 2D-TPP to distinguish proteins that bind to TH6744 in a dose-dependent manner. Overlapping stabilized and destabilized proteins from two TPP analyses show strong enrichment of the host chaperone and co-chaperone network. The most significantly stabilized protein in TPP-TR, co-chaperone DNAJB11, has been described to localize to Dengue virus induced replication complexes in ER promoting vRNA synthesis [51] and to mediate Simian Virus 40 capsid disassembly from the ER [52], illustrating DNAJB11 supportive role for enveloped RNA and non-enveloped DNA viruses. Additionally, we observed uniform stabilization between proteostasis ER residents such as co-chaperones DNAJC13, DNAJC3, EDEM3, chaperones HSP90B1, HYOU1 and CALR and destabilization between cytosolic chaperones such as HSPA1A, HSPA2 and HSPA8. Interestingly, a regulator protein of ERAD, EDEM3 was shown to interact with SARS-Cov2 proteins Orf8 and Orf9b [53]. Our findings imply that TH6744 affects multiple components of a chaperone network which RNA viruses heavily depend on for propagation [54]. Structural similarities between TH3289 and TH6744 and antiviral phenotypes observed with both compounds suggest that TH3289 targeting same host targets as TH6744. Further target identification studies are needed to confirm TH3289’s target proteins.

Inhibiting the host protein folding machinery has been proposed as a promising broad antiviral strategy [55] and during recent years, Hsp70 have been validated as a promising antiviral target [56]. HSP family members possess distinct functions in various steps of RNA and DNA viruses such as supporting CCHFV replication [39]. We thereby chose to validate Hsp70 as one of TH6744 targets. We confirmed previously reported interaction between HAZV NP and Hsp70 [39] and show an increase of Hsp70–HAZV NP interaction after TH6744 treatment. Additionally, we validated dose-dependent thermal destabilization of Hsp70 by TH6744 using CETSA, illustrating how TH6744 sensitizes Hsp70 protein to heat-induced denaturation. Based on thermal destabilization of cytosolic Hsp70 and stabilization of numerous co-chaperones, we propose that TH6744 binds to co-chaperones of Hsp70 multiprotein complexes resulting in Hsp70 thermal destabilization and thereby impairing the function of Hsp70. Increased Hsp70–HAZV NP interaction can reflect higher levels of aggregated HAZV NP that cannot be folded due to high viral protein production and compromised Hsp70 folding capacity. We further suggest that potent reduction of HAZV progeny release after TH6744 treatment is due to reduced Hsp70 folding capacity leading to impaired infectivity while being well tolerated by the host cell. Compounds’ antiviral properties or binding targets have not been optimized and thus their antiviral activity is in micromolar range and they bind to many host proteins, as expected from early hit compounds from phenotypic screening. However, these initial hit compounds can be used as tool compounds to study their antiviral activity and to identify their target proteins revealing new host–target interactions. Collectively, numerous target candidates from TPP remain to be further validated and future studies will provide understanding on the direct and indirect TH6744 targets.

## Supplementary Materials

The following are available online at https://www.mdpi.com/1999-4915/12/12/1423/s1. Figure S1. Quality assessment of the phenotypic antiviral assay and Toxicity of TH3289. Figure S2. TH3289 has a favorable toxicity profile in Vero and Huh7 cell lines. Figure S3. Optimizing TPP conditions and quality control of the analyses. Figure S4. 2D-TPP identifies enrichment of proteostasis pathways by TH6744 treatment. Figure S5. Hsp70 is destabilized after TH6744 treatment. Supplementary Material and methods. Table S1: siRNAs. Table S2: Antibodies. Table S3: DNA Dyes. Data S1. Antiviral screening. Data S2. Dose-response treatment of TH6744 and TH3289. Data S3 is accessible in 10.17044/scilifelab.13089023. Data S4. List of proteins from 2D-TPP analysis in U87 cells (FDR). Data S5. List of proteins from 2D-TPP analysis in U87 cells (Fold-change). Data S6. Uniprot annotation of overlapping TPP-TR and 2D-TPP hits. Data S7. Raw uncropped blots.
