# Supplementary material for: Novel Broad-Spectrum Antiviral Inhibitors Targeting Host Factors Essential for Replication of Pathogenic RNA Viruses
Source: Viruses. 2020 Dec 10;12(12):1423. doi: 10.3390/v12121423 (PMC7762994; doi:10.3390/v12121423)
Supplement: Supplementary file 1 [file viruses-12-01423-s001.zip › Revised Supplementary material 2/Tampere et al_Revised Supplementary material_MT.pdf]

Supplementary Material

Novel broad-spectrum antiviral inhibitors targeting host factors essential for replication of pathogenic RNA viruses

Tampere et al.

This file includes:
Figures S1 - S5
Supplementary Materials and Methods
Tables S1 - S3

Supplementary figures

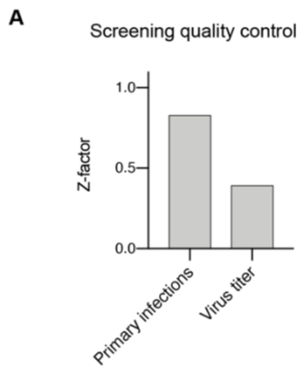

Figure S1. Quality assessment of the phenotypic antiviral assay. (**A**) Z-factors were calculated from primary screening and titer screening.

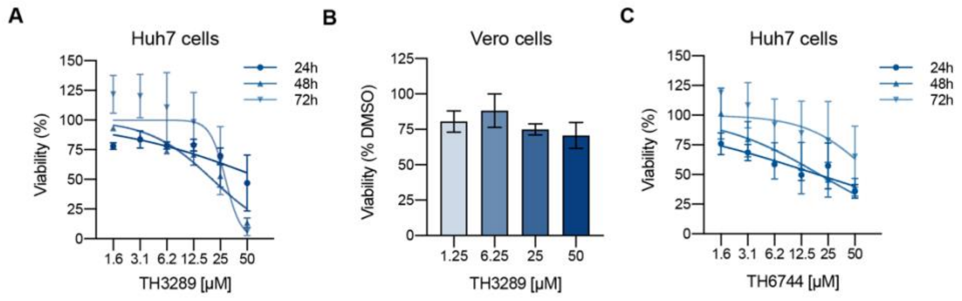

Figure S2. TH3289 has a favourable toxicity profile in Huh7 and Vero cell lines. (**A, C**) Huh7 cells were treated with indicated doses of TH3289 (A) or TH6744 (C) for 24 h (dark blue), 48 h (middle blue) or 72 h (light blue). Cellular viability was measured by resazurin assay. Data is normalized to DMSO control and presented as a mean ± SD from n=3 biological replicates. Curve fitting was performed to determine IC_{50} using a sigmoidal, 4PL model in GraphPadPrism. (**B**) Vero cells were treated with increasing doses of TH3289 for 48 h followed by viability measurements by MTT assay. Data is presented as Mean ± SD from n=2 biological replicates.

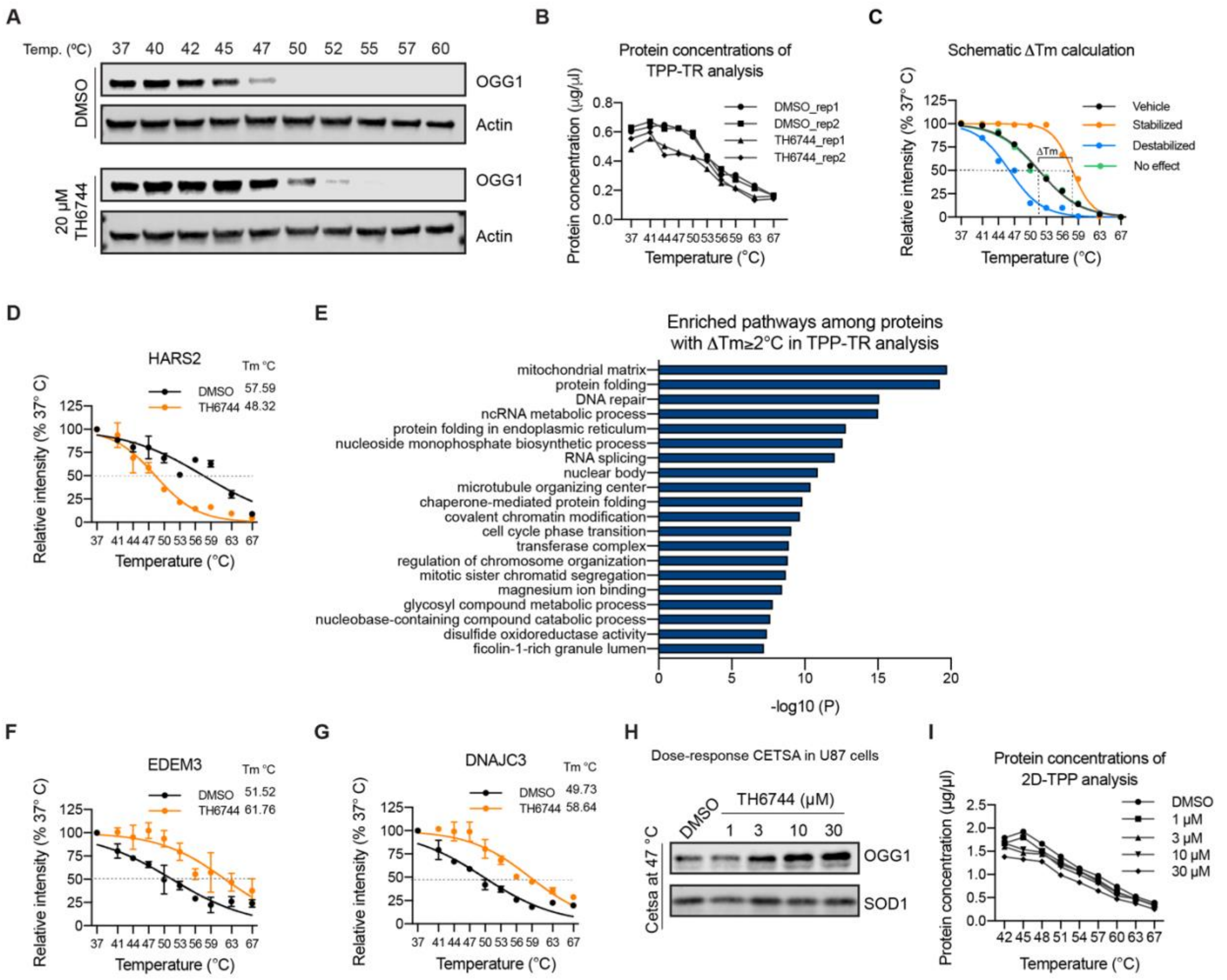

Figure S3. Optimizing TPP conditions and quality control of the analyses. (**A**) CETSA-TR in living SW13 cells. SW13 cells were treated with 20 µM TH6744 for 2 hours followed by CETSA at indicated temperatures and western blot. Data is representative of n=3 biological replicates. (**B**) Total protein concentrations of TPP-TR samples from SW13 cells treated with DMSO or 20 µM TH6744 for 4 hours and analyzed by BCA Protein Assay kit. (**C**) Visual explanation of protein melting difference ΔTm calculation. ΔTm is calculated as a difference in protein melting point Tm between DMSO and TH6744 treated samples. (**D, F, G**) TPP-TR melt curves of HARS2 (**D**), EDEM3 (**F**) and DNAJC3 (**G**) proteins. Normalized relative protein intensities from TPP-TR were fitted using sigmoidal 4-parameter curve fitting. Data is expressed as mean ± SD from n=2 technical replicates. (**E**) Top 20 enriched terms clusters across TPP-TR hits with ΔTm >2C. Individual terms were grouped based on the p-value <0.01, a minimum count of 3 and an enrichment factor >1.5 using Metascape gene annotation resource. (**H**) Isothermal TH6744 dose-response CETSA at 47 °C in living U87 cells. U87 cells were treated with indicated TH6744 doses for 1 hour followed by CETSA at constant temperature 47°C and western blotting. Data of n=1 biological replicate is presented. (**I**) Total protein concentrations of 2D-TPP samples from U87 cells treated with DMSO or indicated TH6744 doses for 1.5 hours and analyzed by DC Protein Assay kit.

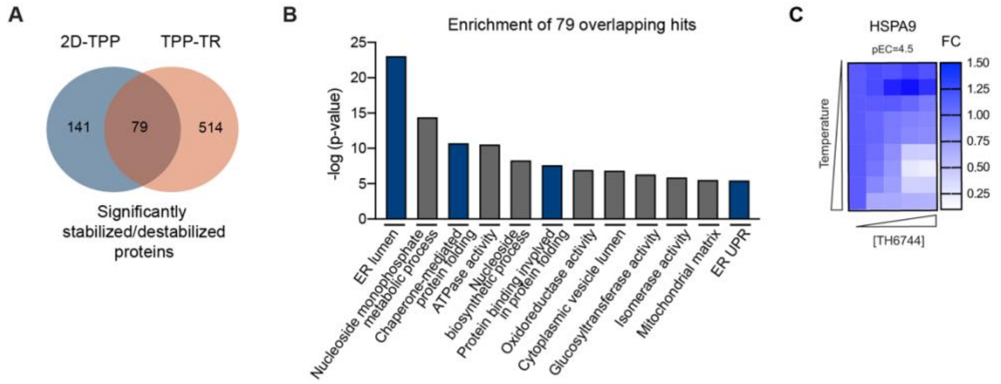

Figure S4. 2D-TPP identifies enrichment of proteostasis pathways by TH6744 treatment. (**A**) Venn diagram showing an overlap between the TPP-TR hits (593 proteins identified by selection criteria and in addition ΔTm>2℃ threshold) and 2D-TPP hits (220 proteins identified at 1% false discovery rate and F-statistic >15 threshold). (**B**) Top 12 enriched term clusters across 79 proteins overlapping between TPP-TR and 2D-TPP. Individual terms were grouped based on the p-value <0.01, a minimum count of 3 and an enrichment factor >1.5 using Metascape gene annotation resource. (**C**) Heat map showing relative HSPA9 protein abundance as fold change of TH6744 treatment (X-axis, 1 μM, 3 μM, 10 μM and 30 μM) compared to DMSO treatment (first column in the graph) in presence of increasing temperatures (Y-axis, 42, 45, 48, 51, 54, 57, 60, 63 and 67 °C). pEC_{50} (pEC_{50}= $-log(EC_{50})$ illustrates binding affinity of TH6744 to host targets. n=1 biological replicate.

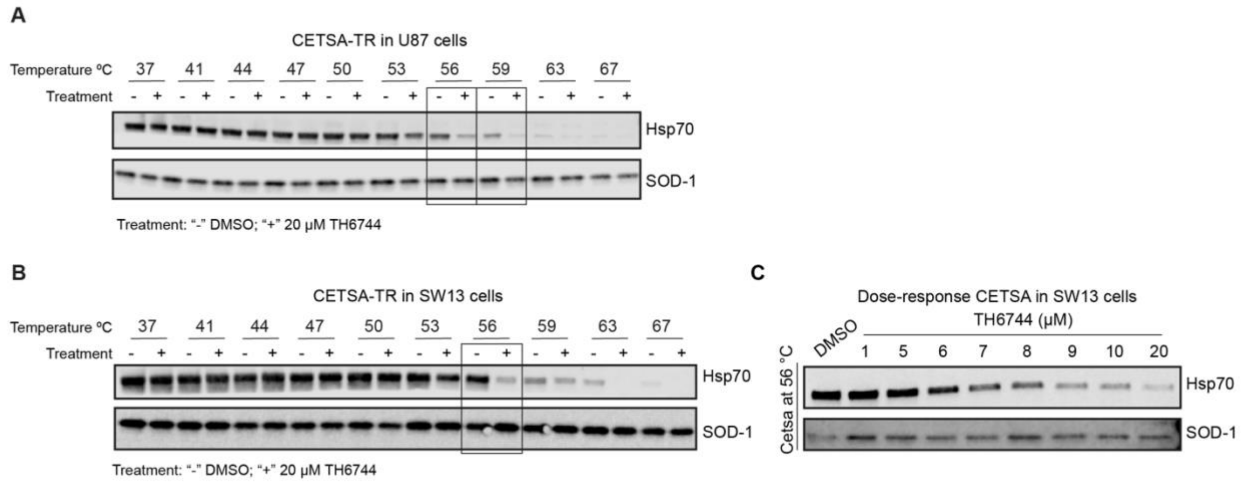

Figure S5. Hsp70 is destabilized after TH6744 treatment. (**A**) CETSA-TR in living U87 cells. U87 cells were treated with 20 μM TH6744 for 4 hours followed by CETSA at indicated temperatures. Data is representative of n=2 biological replicates. (**B**) CETSA-TR in living SW13 cells. SW13 cells were treated with 20 μM TH6744 for 4 hours followed by CETSA at indicated temperatures. Data is representative of n=3 biological replicates. (**C**) Isothermal TH6744 dose-

response CETSA at 56°C in living SW13 cells. SW13 cells were treated with indicated TH6744 doses for 4 hours followed by CETSA at constant temperature 56°C. Data is representative of n=2 biological replicates.

Supplementary Material and methods

Chemistry

All the reagents were commercially available and were used without further purification. Analytical thin-layer chromatography was performed on silica gel 60 F-254 plates (E. Merck) and visualized with UV light. Purification of compounds by flash chromatography was achieved using a Biotage SP4 system. NMR spectra were recorded on a Bruker 400 MHz system. The chemical shifts for ^{1}H are referenced via residual solvent signal. Liquid Chromatography Mass Spectrometry (LC-MS) experiments to determine retention times (RT) and associated mass ions were performed on an Agilent MSD mass spectrometer connected to an Agilent 1100 system with: System A: Column ACE 3 C8, 3 µm, 50 x 3.0 mm maintained at 40°C. 0.1% (v/v) TFA in water (A) and MeCN (B) were used as mobile phases at a flow rate of 1 mL/min, with a gradient time of 3.0 min. System B: Column Xterra MS C18, 3.5 µm, 50 x 3.0 mm maintained at 40°C. Water (containing 10 mM NH_4HCO_3; pH = 10, A) and MeCN (B) were used as mobile phases at a flow rate of 1 mL/min, with a gradient time of 3.0 min. Preparative HPLC was performed on a Gilson HPLC system. System A: small quantities, column ACE C8, 5 µm (50 x 21.2 mm); large quantities, column ACE C8, 5 µm (150 x 30 mm); 0.1% TFA (v/v) in H2O and MeCN were used as mobile phases at a flow rate of 30 or 38 mL/min (for small and large quantity respectively) with a gradient time of 7 min. System B: small quantities, column XTerra Prep MS C18, 5 µm OBD (19 x 50 mm); large quantities, column XBridge Prep C18, 5 µm CBD (30 x 75 mm); H_2O (containing 50 mM NH_4HCO_3; pH = 10) and MeCN were used as mobile phases at a flow rate of 45 mL/min, with a gradient time of 11 min.

Synthesis of 4-(5-chloro-2-oxo-2,3-dihydro-1H-benzo[d]imidazol-1-yl)-N-(4-iodophenyl)piperidine-1-carboxamide TH3289

A mixture of 5-chloro-1-(4-piperidyl)-1H-benzimidazol-2-one (30 mg, 0.12 mmol) and 4-iodophenyl isocyanate (29 mg, 0.12 mmol) was stirred in DCM (3 mL) at r.t. for 3 h. The precipitate was collected by filtration and was washed sequentially with DCM, water, and methanol to give the title compound as a white solid (45 mg, 76%). LCMS $[M+H]^+$ 497; ^{1}H NMR (400 MHz, DMSO-d_6) δ ppm 11.05 (br. s., 1 H) 8.69 (s, 1 H) 7.52 - 7.59 (m, 2 H) 7.32 - 7.39 (m, 2 H) 7.24 (d, *J* = 8.37 Hz, 1 H) 6.97 - 7.06 (m, 2 H) 4.33 - 4.44 (m, 1 H) 4.23 - 4.33 (m, 2 H) 2.86 - 2.99 (m, 2 H) 2.15 - 2.31 (m, 2 H) 1.67 - 1.80 (m, 2 H); ^{13}C NMR (100 MHz, DMSO-d_6) δ ppm 154.4, 153.6, 140.7, 136.9, 129.5, 128.3, 124.8, 121.7, 120.1, 109.7, 108.7, 84.6, 50.3, 43.4, 28.6.

Synthesis of ethyl 1-(1-(1-((4-iodophenyl)carbamoyl)piperidin-4-yl)-2-oxo-2,3-dihydro-1H-benzo[d]imidazol-4-yl)-1H-pyrazole-4-carboxylate TH6744

Step 1: ethyl 1-(3-fluoro-2-nitrophenyl)-1H-pyrazole-4-carboxylate

A mixture of ethyl 1H-pyrazole-4-carboxylate (1.40 g, 10.0 mmol) and sodium hydride (0.440 g, 11.0 mmol; as a 60% dispersion in mineral oil) was stirred in THF (20 mL) at r.t. for 20 min. To the mixture was then added 1,3-difluoro-2-nitrobenzene (1.59 g, 10.0 mmol) and the temperature was raised to 110°C and stirring continued for 16 h. Purification by silica gel flash chromatography afforded ethyl 1-(3-fluoro-2-nitrophenyl)-1H-pyrazole-4-carboxylate (1.50 g, 54%). LCMS $[M+H]^+$ 280. ^{1}H NMR (400 MHz, $CDCl_3$) δ ppm 8.19 (s, 1 H), 8.03 (s, 1 H), 7.56 (app. td, *J* = 8.4, 5.5 Hz, 1 H), 7.32 (dt, *J* = 8.3, 1.2 Hz, 1 H), 7.28 (app. td, *J* = 8.7, 1.2 Hz, 1 H), 7.13 (q, *J* = 7.1 Hz, 2 H), 1.30 (t, *J* = 7.1 Hz, 3 H).

Step 2: tert-butyl 4-((3-(4-(ethoxycarbonyl)-1H-pyrazol-1-yl)-2-nitrophenyl)amino)piperidine-1-carboxylate

A mixture of ethyl 1-(3-fluoro-2-nitro-phenyl)pyrazole-4-carboxylate (1.00 g, 3.58 mmol), tert-butyl 4-aminopiperidine-1-carboxylate (0.789 g, 3.94 mmol) and N,N-diisopropylethylamine (0.686 mL, 3.94 mmol) was stirred in 2-propanol (10 mL) at 120 °C for 16 h. Purification by silica gel flash chromatography afforded tert-butyl 4-((3-(4-(ethoxycarbonyl)-1H-pyrazol-1-yl)-2-nitrophenyl)amino)piperidine-1-carboxylate (1.65 g, 87%). LCMS $[M+H]^+$ 460. ^{1}H NMR (400 MHz, $CDCl_3$) δ ppm 8.19 (d, *J* = 0.4 Hz, 1 H), 8.08 (d, *J* = 0.4 Hz, 1 H), 7.43 (dd, *J* = 8.7, 7.7 Hz, 1 H), 6.95 (br. d, *J* = 8.3 Hz, 1 H), 6.73 (dd, *J* = 7.7, 1.0 Hz, 1 H), 4.35 (q, *J* = 7.1 Hz, 2 H), 4.01 – 4.08 (m, 2 H), 3.59 – 3.67 (m, 1 H), 2.99 – 3.07 (m, 2 H), 2.02 – 2.09 (m, 2 H), 1.47 – 1.56 (overlapping m, 2 H), 1.49 (s, 9 H), 1.39 (t, *J* = 7.1 Hz, 3 H).

Step 3: tert-butyl 4-(4-(4-(ethoxycarbonyl)-1H-pyrazol-1-yl)-2-oxo-2,3-dihydro-1H-benzo[d]imidazol-1-yl)piperidine-1-carboxylate

To a stirred mixture of tert-butyl 4-[3-(4-ethoxycarbonylpyrazol-1-yl)-2-nitro-anilino]piperidine-1-carboxylate (1.41 g, 3.07 mmol) and $NiCl_2$ (0.080 g, 0.61 mmol) in acetonitrile/water (25 mL, 9:1) at r.t. was slowly added $NaBH_4$ (0.464 g, 12.3 mmol). The mixture was stirred for 20 min and then transferred to an extraction funnel and extracted (DCM/sat. $NaHCO_3$), dried over $MgSO_4$ and filtered. To the collected organic layer was then added bis(trichloromethyl) carbonate (0.317 g, 1.07 mmol) followed by N,N-diisopropylethylamine (1.34 mL, 7.66 mmol) and the resulting solution was stirred at r.t. for 10 min. Purification by silica gel flash chromatography afforded tert-butyl 4-(4-(4-(ethoxycarbonyl)-1H-pyrazol-1-yl)-2-oxo-2,3-dihydro-1H-benzo[d]imidazol-1-yl)piperidine-1-carboxylate (0.610 g, 44%). LCMS $[M+H]^+$ 456. ^{1}H NMR (400 MHz, DMSO-d_6) δ ppm 10.86 (s., 1 H), 8.91 (d, *J* = 0.5 Hz, 1 H), 8.13 (d, *J* = 0.5 Hz, 1 H), 7.41 (dd, *J* = 8.2, 0.5 Hz, 1 H), 7.30 (br. d, *J* = 7.8 Hz, 1 H), 7.13 (t, *J* = 8.1 Hz, 1 H), 4.40 (tt, *J* = 12.1, 3.9 Hz, 1 H), 4.29 (q, *J* = 7.1 Hz, 2 H), 4.05 – 4.18 (m, 2 H), 2.80 – 2.99 (m, 2 H), 2.18 – 2.30 (m, 2 H), 1.69 – 1.77 (m, 2 H), 1.45 (s, 9 H), 1.32 (t, *J* = 7.1 Hz, 3 H). ^{13}C NMR (100 MHz, DMSO-d_6) δ ppm 162.5, 154.3, 153.9, 142.0, 133.2, 131.7, 122.6, 121.4, 121.1, 116.1, 114.0, 108.3, 79.3, 60.4, 50.8, 43.1 (br. s), 28.9 (br. s), 28.6, 14.8.

Step 4: ethyl 1-(2-oxo-1-(piperidin-4-yl)-2,3-dihydro-1H-benzo[d]imidazol-4-yl)-1H-pyrazole-4-carboxylate

A solution of tert-butyl 4-(4-(4-(ethoxycarbonyl)-1H-pyrazol-1-yl)-2-oxo-2,3-dihydro-1H-benzo[d]imidazol-1-yl)piperidine-1-carboxylate (0.384 g, 0.842 mmol) in trifluoroacetic acid (2 mL) was stirred at r.t. for 10 min and then concentrated to give ethyl 1-(2-oxo-1-(piperidin-4-yl)-2,3-dihydro-1H-benzo[d]imidazol-4-yl)-1H-pyrazole-4-carboxylate as a trifluoroacetate salt (0.390 g, quant.). LCMS $[M+H]^+$ 356. ^{1}H NMR (400 MHz, DMSO-d_6) d ppm 10.95 (s, 1 H), 8.94 (s, 1 H), 8.69 (br. s., 1 H), 8.46 (br. s., 1 H), 8.15 (s, 1 H), 7.46 (d, *J* = 7.9 Hz, 1 H), 7.39 (d, *J* = 7.6 Hz, 1

H), 7.16 - 7.23 (m, 1 H), 4.54 - 4.64 (m, 1 H), 4.29 (q, *J* = 7.0 Hz, 2 H), 3.42 - 3.51 (m, 2 H), 3.04 - 3.18 (m, 2 H), 2.54 - 2.69 (m, 2 H), 1.86 - 1.97 (m, 2 H), 1.32 (t, *J* = 7.0 Hz, 3 H).

Step 5: ethyl 1-(1-(1-((4-iodophenyl)carbamoyl)piperidin-4-yl)-2-oxo-2,3-dihydro-1H-benzo[d]imidazol-4-yl)-1H-pyrazole-4-carboxylate (TH6744)

To a stirred mixture of ethyl 1-(2-oxo-1-(piperidin-4-yl)-2,3-dihydro-1H-benzo[d]imidazol-4-yl)-1H-pyrazole-4-carboxylate trifluoroacetate (0.300 g, 0.639 mmol) and N,N-diisopropylethylamine (0.223 mL, 1.28 mmol) in dichloromethane (30 mL) was added a solution of 4-iodophenyl isocyanate (0.157 g, 0.639 mmol) in dichloromethane (2 mL). The mixture was stirred at r.t. for 2 h and the solid material was collected by filtration and washed with water followed by dichloromethane to afford the title compound as a white solid (0.300 g, 78%). LCMS $[M+H]^+$ 601; ^{1}H NMR (400 MHz, DMSO-d_6) δ ppm 10.88 (br. s., 1 H) 8.92 (s, 1 H) 8.71 (s, 1 H) 8.13 (s, 1 H) 7.54 - 7.60 (m, 2 H) 7.29 - 7.44 (m, 4 H) 7.13 (t, *J* = 8.13 Hz, 1 H) 4.40 - 4.56 (m, 1 H) 4.23 - 4.35 (m, 4 H) 2.85 - 3.06 (m, 2 H) 2.21 - 2.42 (m, 2 H) 1.71 - 1.85 (m, 2 H) 1.31 (t, *J* = 7.11 Hz, 3 H); ^{13}C NMR (100 MHz, DMSO-d_6) δ ppm 162.1, 154.4, 153.5, 141.6, 140.7, 136.9, 132.7, 131.3, 122.1, 121.7, 120.9, 120.6, 115.6, 113.5, 107.8, 84.5, 60.0, 50.5, 43.5, 28.6, 14.3.

Synthesis of 4-(1H-indol-3-yl)-N-(4-iodophenyl)piperidine-1-carboxamide TH4448

To a solution of 3-(piperidin-4-yl)-1H-indole (15 mg, 0.075 mmol) and triethylamine (21 µL, 0.15 mmol) in DCM (2 mL) was added 4-iodophenyl isocyanate (20 mg, 0.082 mmol). The mixture was stirred at room temperature for 12 h. The precipitate was collected by filtration and washed with cold dichloromethane to give the desired product as a white solid (24 mg, 72%). LCMS [M+H]+ 497; ^{1}H NMR (400 MHz, DMSO-d6) δ ppm 10.79 (br. s., 1 H) 8.62 (s, 1 H) 7.50 - 7.62 (m, 3 H) 7.30 - 7.40 (m, 3 H) 7.10 - 7.14 (m, 1 H) 6.91 - 7.09 (m, 2 H) 4.18 - 4.32 (m, 2 H) 2.88 - 3.08 (m, 3 H) 1.89 - 2.08 (m, 2 H) 1.50 - 1.69 (m, 2 H); ^{13}C NMR (100 MHz, DMSO-d_6) δ ppm 154.6, 140.8, 136.8, 136.3, 126.2, 121.7, 120.9, 120.7, 119.2, 118.5, 118.1, 111.4, 84.4, 44.5, 33.1, 32.7.

Supplementary Tables

Table S1: siRNAs

Product	siRNA target	Sequence	Pool cat no	Individual cat no	Provider
OGG1 pooled siRNA	OGG1	CGA CAA GAC CCC AUC GAA U	L-005147-00	J-005147-09	Dharmacon
OGG1 pooled siRNA	OGG1	GGA CAA UCU UUC CGG UGGA	L-005147-00	J-005147-10	Dharmacon

OGG1 pooled siRNA	OGG1	GCU CAG AAA UUC CAA GGUG	L-005147-00	J-005147-11	Dharmacon
OGG1 pooled siRNA	OGG1	UAC CCU GGC UCA ACU GUAU	L-005147-00	J-005147-12	Dharmacon
OGG1 siRNA_1	OGG1	UCC CUG GAC UCC CAC UUC CAA			Dharmacon
OGG1 siRNA_2	OGG1				
ON-TARGETplus Non-targeting Pool	Neg control	UGG UUU ACA UGU CGA CUAA		D-001810-10	Dharmacon
AllStars Hs Cell Death Control	Pos control				Qiagen

Table S2: Antibodies

**Target**	**Host**	**Company**	**Cat. Number**	**Dilution**
HAZV NP	Mouse	in-house production		1:2000
Sars-CoV-2 Spike	Mouse	GeneTex	GTX632604	1:1000
Coronavirus pan Monoclonal Antibody (FIPV3-70)	Mouse	ThermoFisher Scientific	MA1-82189	1:500

CCHFV NP	Rabbit	in-house production		1:150
EBOV	Mouse	in-house production		1:100
OGG1	Rabbit	Abcam	ab93670	1:1000
SOD1	Mouse	Santa Cruz	sc-17767	1:2000
hbActin	Mouse	Abcam	ab6276	1:3000
HSP70	Rabbit	Abcam	ab45133	1:2000
Donkey anti-Mouse IgG (H+L) Highly Cross-Adsorbed Secondary Antibody, Alexa Fluor 488	Donkey	ThermoFisher Scientific	A-21202	1:800
Goat anti-Mouse IgG (H+L) Cross-Adsorbed Secondary Antibody, Alexa Fluor 647	Goat	ThermoFisher Scientific	A-21235	1:800
Goat anti-Rabbit IgG (H+L) Highly Cross-Adsorbed Secondary Antibody, Alexa Fluor 488	Goat	ThermoFisher Scientific	A11008	1:800
IRDye 680RD Donkey anti-mouse	Donkey	Licor	926-68072	1:500
IRDye® 680RD Donkey anti rabbit	Donkey	Licor	926-68073	1:500
IRDye® 800CW Donkey anti-Mouse IgG	Donkey	Licor	926-32212	1:500
IRDye® 800CW Donkey anti-Rabbit IgG	Donkey	Licor	926-32213	1:500

Table S3: DNA Dyes

Dye	Company	Cat Number	Dilution

DAPI (4',6-Diamidino-2-Phenylindole, Dihydrochloride)	ThermoFisher Scientific	D1306	1:1000
