# Supplementary material for: Novel Broad-Spectrum Antiviral Inhibitors Targeting Host Factors Essential for Replication of Pathogenic RNA Viruses
Source: Viruses. 2020 Dec 10;12(12):1423. doi: 10.3390/v12121423 (PMC7762994; doi:10.3390/v12121423)
Supplement: Supplementary file 1 [file viruses-12-01423-s001.zip › Revised Supplementary material 2/Data S7_raw blots.pdf]

Figure 3d

Anti OGG1

Anti SOD1

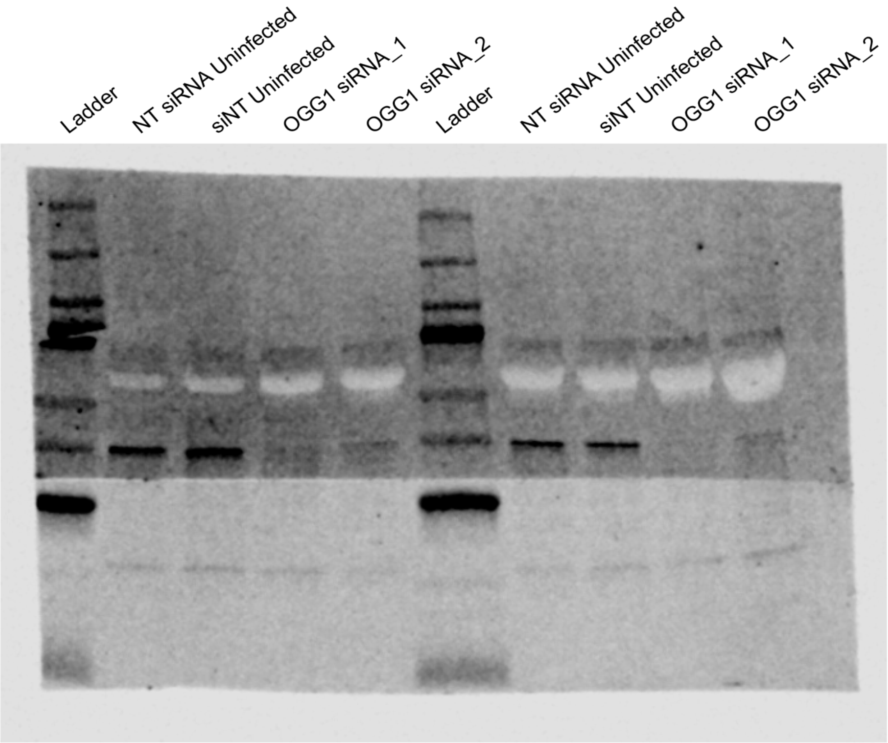

Anti OGG1

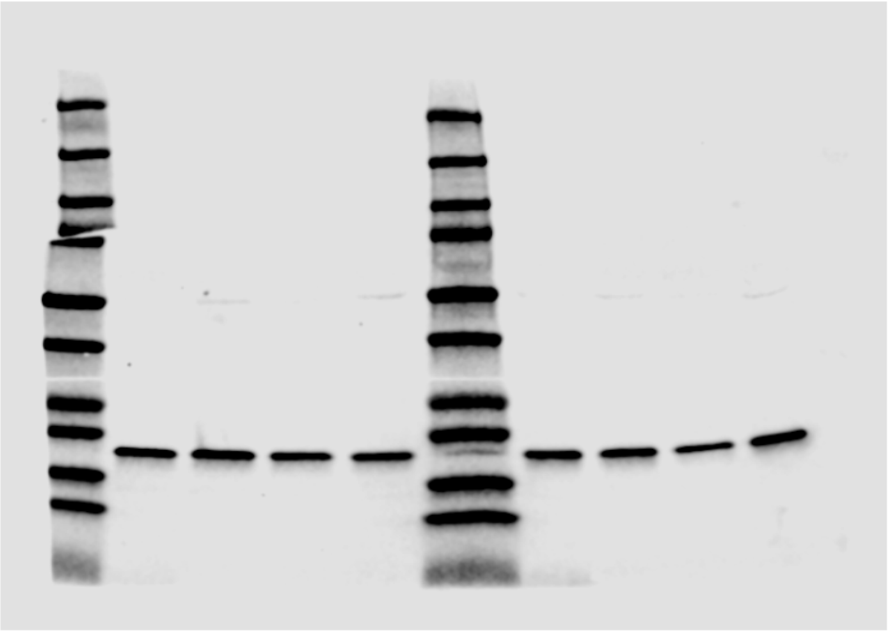

Anti SOD1

Figure 6a
Anti Hsp70
Anti Actin

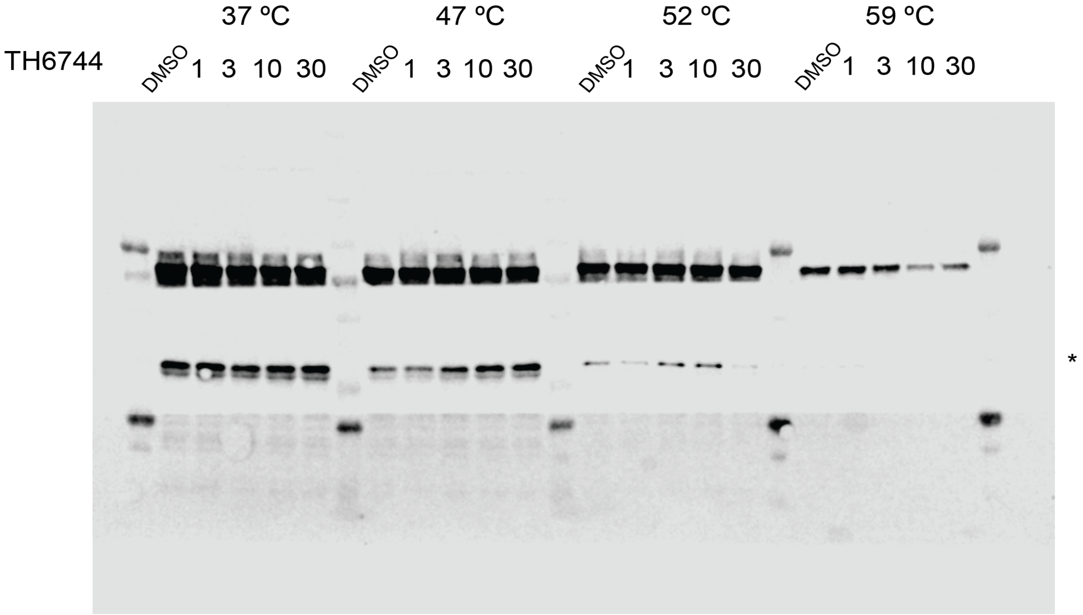

Anti Hsp70 *Anti OGG1

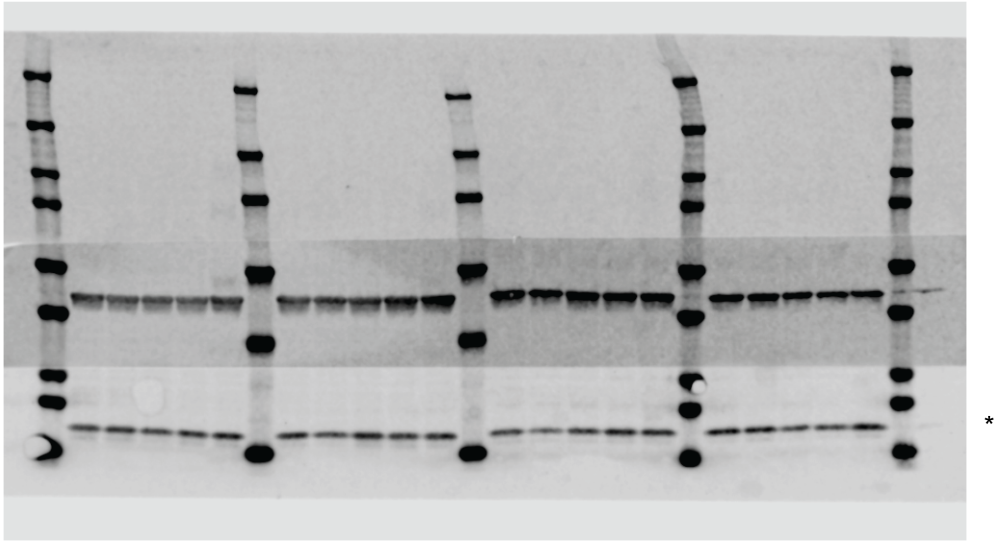

Anti Actin *Anti SOD1

Figure 6b
Anti Hsp70
Anti HAZV NP

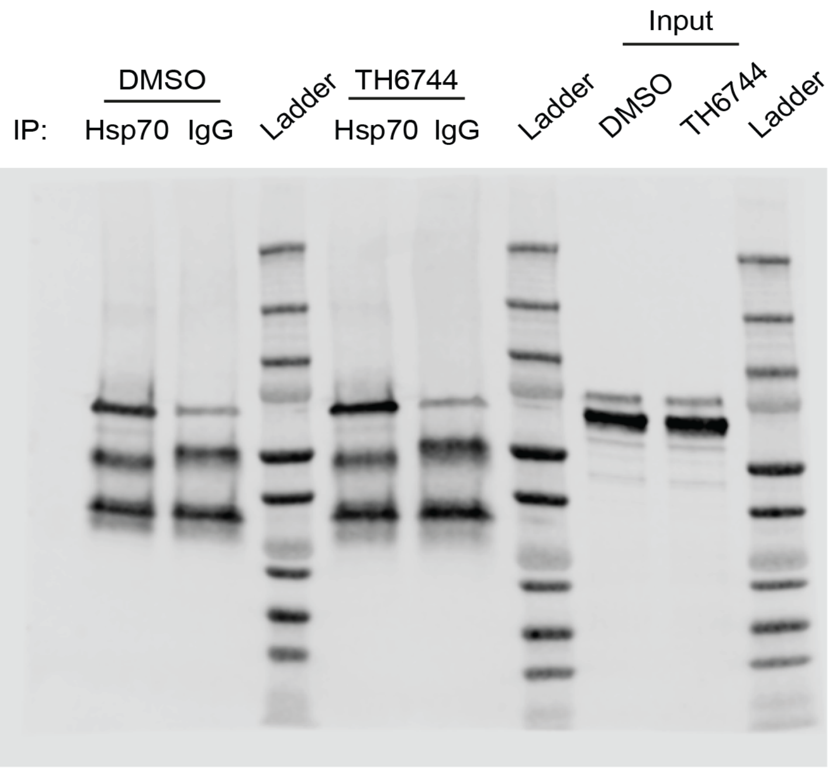

Anti Hsp70

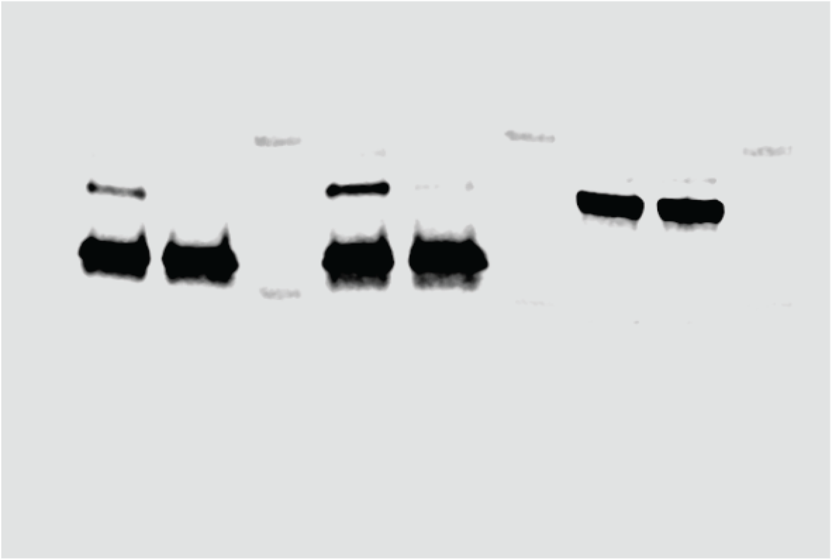

Anti HAZV NP

Figure S3a
Anti OGG1
Anti Actin

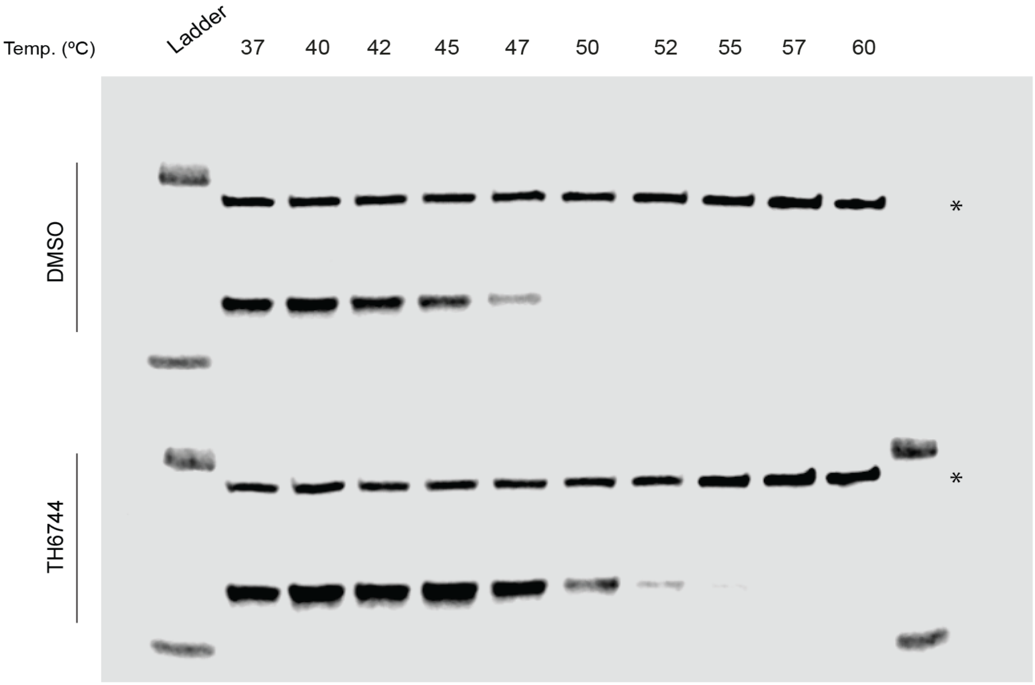

Anti OGG1

* Unspecific signal

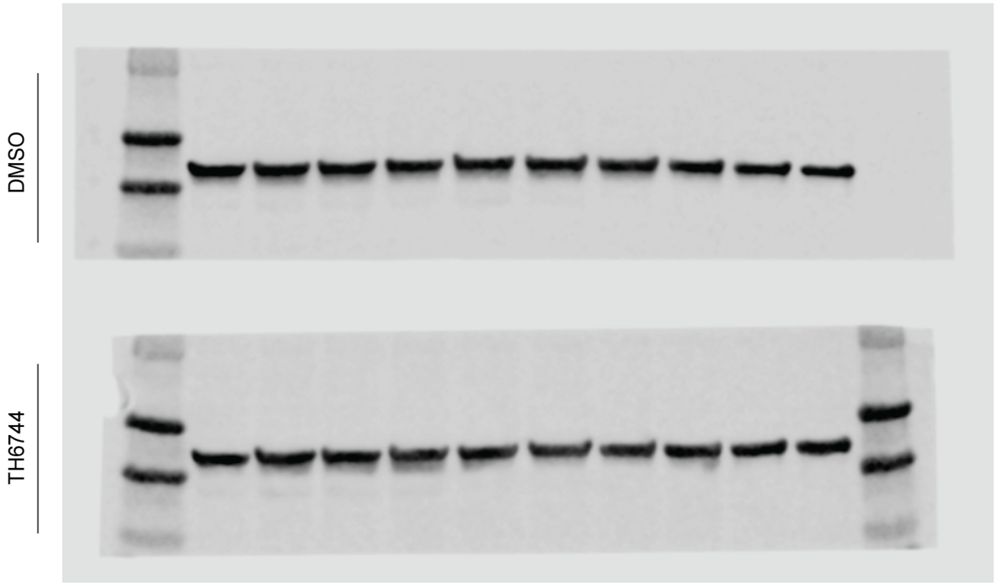

Anti Actin

Figure S3h

Anti OGG1

Anti SOD1

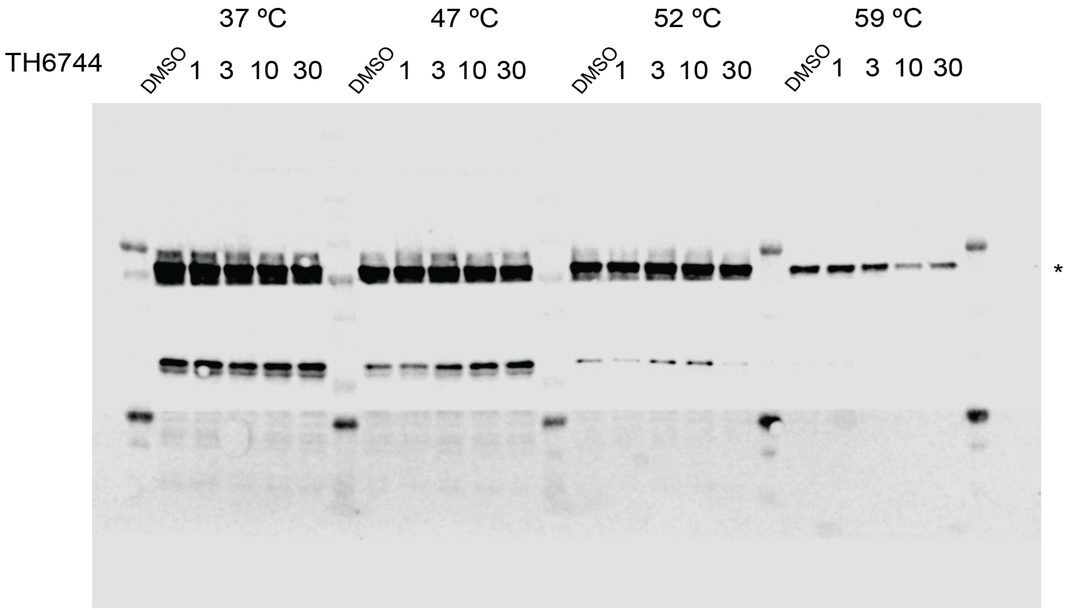

Anti OGG1 *Anti Hsp70

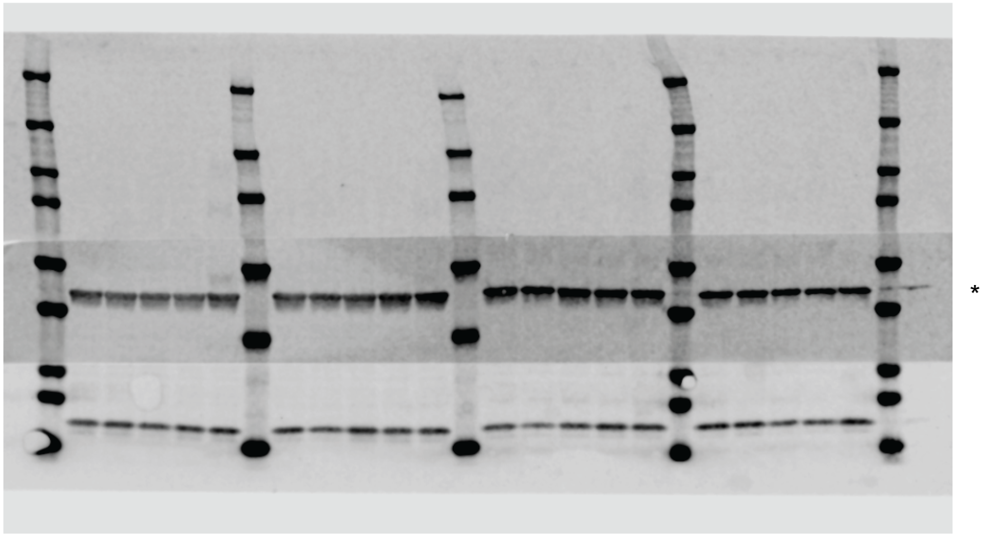

Anti SOD1 *Anti Actin

Figure S5a

Anti Hsp70

Anti SOD1

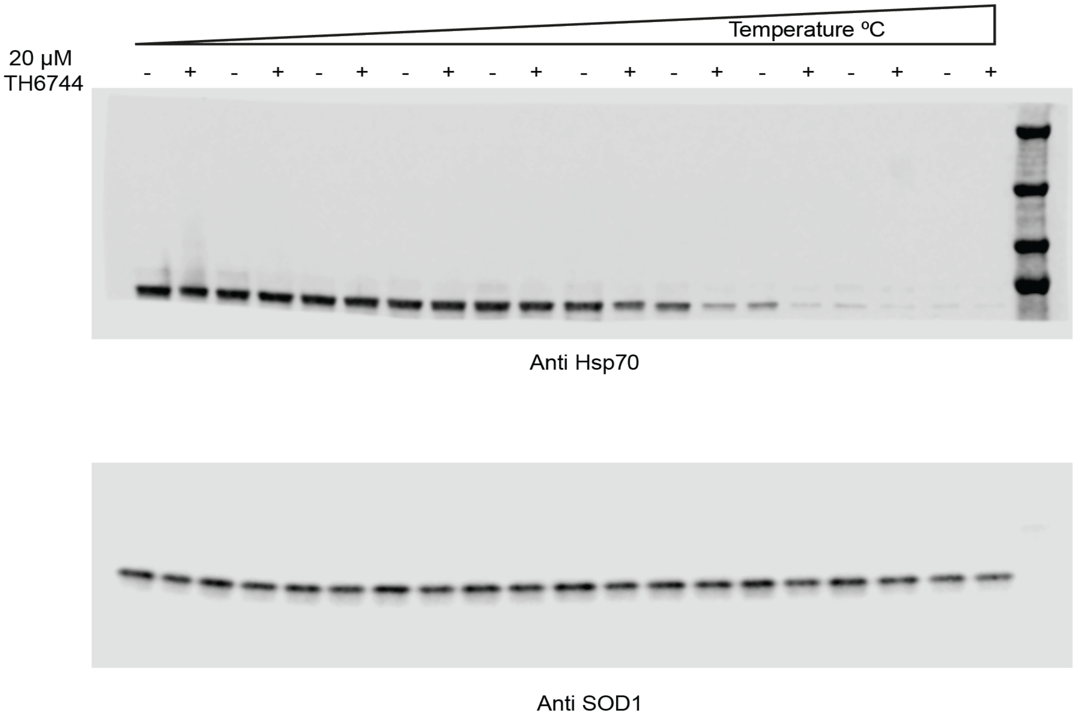

Figure S5b

Anti Hsp70

Anti SOD1

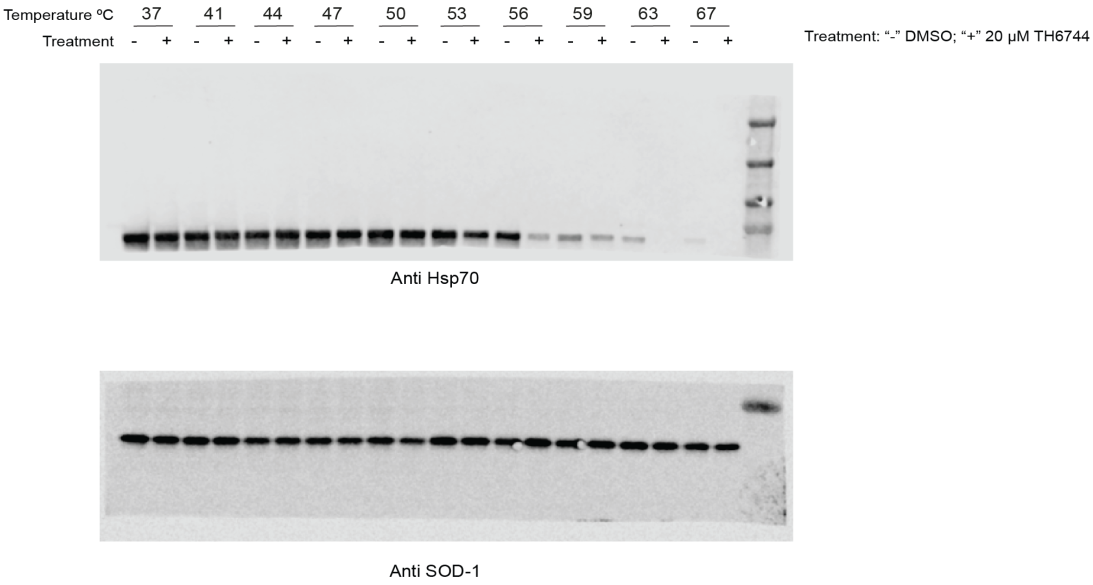

Figure S5c
Anti Hsp70
Anti SOD1

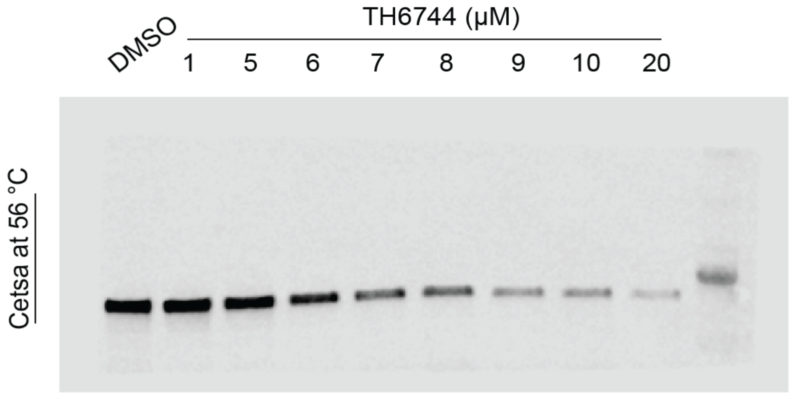

Anti Hsp70

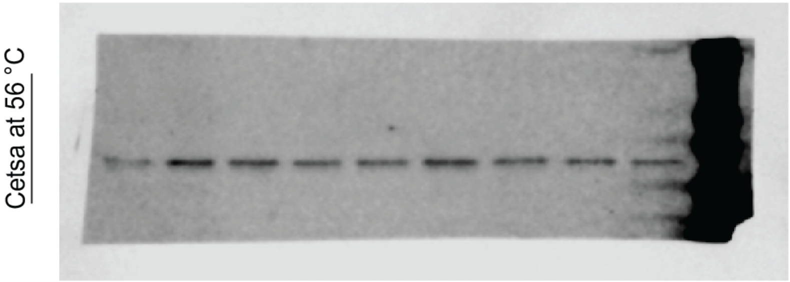

Anti SOD-1
